# *Ex vivo* live cell tracking in kidney organoids using light sheet fluorescence microscopy

**DOI:** 10.1371/journal.pone.0199918

**Published:** 2018-07-26

**Authors:** Marie Held, Ilaria Santeramo, Bettina Wilm, Patricia Murray, Raphaël Lévy

**Affiliations:** 1 Institute of Integrative Biology, University of Liverpool, Liverpool, United Kingdom; 2 Institute of Translational Medicine, University of Liverpool, Liverpool, United Kingdom; National Cancer Institute, UNITED STATES

## Abstract

Screening cells for their differentiation potential requires a combination of tissue culture models and imaging methods that allow for long-term tracking of the location and function of cells. Embryonic kidney re-aggregation *in vitro* assays have been established which allow for the monitoring of organotypic cell behaviour in re-aggregated and chimeric renal organoids. However, evaluation of cell integration is hampered by the high photonic load of standard fluorescence microscopy which poses challenges for imaging three-dimensional systems in real-time over a time course. Therefore, we employed light sheet microscopy, a technique that vastly reduces photobleaching and phototoxic effects. We have also developed a new method for culturing the re-aggregates which involves immersed culture, generating organoids which more closely reflect development *in vivo*. To facilitate imaging from various angles, we embedded the organoids in a freely rotatable hydrogel cylinder. Endpoint fixing and staining were performed to provide additional biomolecular information. We succeeded in imaging labelled cells within re-aggregated kidney organoids over 15 hours and tracking their fate while simultaneously monitoring the development of organotypic morphological structures. Our results show that Wt1-expressing embryonic kidney cells obtained from transgenic mice could integrate into re-aggregated chimeric kidney organoids and contribute to developing nephrons. Furthermore, the nascent proximal tubules that formed in the re-aggregated tissues using the new culture method displayed secretory function, as evidenced by their ability to secrete an organic anion mimic into the tubular lumen.

## Introduction

Culture systems for growing intact kidney rudiments *ex vivo*, first established by Grobstein in the 1950s, are excellent tools for understanding various aspects of kidney development [1, 2]. In 2002, Atala’s group showed that dissociated bovine kidney rudiments had the remarkable ability to re-aggregate and generate nephron-like structures following subcutaneous implantation into adult cows [3]. More recently, it has been shown that disssociated mouse kidney rudiments can re-aggreate and generate functional nephrons and ureteric buds when cultured *ex vivo* [4–6]. These re-aggreated rudiments are valuable tools for determining the nephrogenic potential of different types of stem or progenitor cells, because the stem/progenitor cell type under investigation can be labelled and then mixed with the dissociated embryonic kidney cells to generate a chimeric re-aggregate [6–10]. The recombinant kidney organ culture involves the isolation of the intact, developing organ, its dissociation and re-aggregation of the cell mixture, which is then typically cultured at the air-liquid interface atop a micro-porous filter.

In the developing kidney, reciprocal inductive interactions between the ureteric bud and metanephric mesenchyme lead to the formation of the metanephric kidney [11, 12]. One of the key transient nephrogenic structures is the cap mesenchyme which, as a morphologically distinguishable layer 4–5 cells deep, is essential for inducing ureteric bud branching as well as providing a source of progenitor cells, ultimately giving rise to the whole nephron, including glomeruli and renal tubules [13, 14]. Any system mimicking renal development should both closely resemble *in vivo* organogenesis, including the formation of important nephrogenic structures, and allow for close monitoring of events. Unfortunately, the re-aggregation assay, though reproducible, results in the generation of organoids that differ in size and organisation. This makes it difficult to generate and compare quantitative data between experiments. Longitudinal monitoring of a single experiment would therefore benefit our understanding of cellular and molecular events during kidney development. Furthermore, cell tracking can help us to answer previously unanswered questions. For instance, what proportion of the labelled cells survives, and when does cell death occur? Do the surviving cells integrate into developing structures? If so, do they actively migrate to developing structures? Previous studies have shown that various types of stem and progenitor cells can integrate into developing structures within chimeric re-aggregates *ex vivo* and can also exhibit function, but it is not known how and when they reach the respective renal structures [6, 7, 9, 15].

Previously, ureteric bud tip cells have been tracked manually in single focal plane confocal images [16]. Lindström et al. [17] performed time-lapse capture of developing nephrons of a signalling reporter mouse strain but quantitative analysis was undertaken following fixation and immunofluorescent staining. Finally, Saarela at al. [18] recorded z-stacks of mouse embryonic kidney organoids repeatedly for up to 20 mins. Automated cell tracking of ureteric bud cells was however performed on single focal plane images.

Re-aggregated tissues cultured at the air-liquid interface grow/self-assemble primarily in two dimensions with little increase in depth [5]. While a flat tissue is advantageous for standard optical microscopy imaging of simulated early kidney development, it does not accurately reflect kidney development *in vivo*, as the developing kidneys *in situ* are spheroidal structures. An organoid context would mimic the physiological situation better but poses greater imaging difficulties. For end point imaging, organoids/organ tissue spheroids are often cultured, fixed, sectioned, stained, and imaged [19]. In such conditions, the three-dimensional morphological and general context are maintained during culture but lost for imaging.

Light sheet fluorescence microscopy [20] allows for the optical sectioning of the sample while maintaining its three-dimensional architecture, i.e. whole mount analysis [20–23]. The imaging system (Zeiss Z.1 Lightsheet) combines two-dimensional illumination with orthogonal camera-based detection. A cylindrical lens shapes the laser light into a thin sheet of light directed onto the sample, illuminating only a section. The camera-based detector records the whole focal plane at once, resulting in fast imaging times. With only a section of the sample being illuminated at any time and rapid frame-wise data capture, light sheet fluorescence microscopy creates a photonic load several orders of magnitude lower than standard confocal fluorescence imaging [20], therefore allowing the capture of transient phenomena [16–18, 24, 25]. Samples are suspended in a water-based gel and can be rotated freely around one axis and therefore imaged from various angles. For live imaging, the samples in the hydrogel are surrounded by growth medium and a controlled environment (temperature, CO_2_ concentration). Cell spheroids have repeatedly been imaged using light sheet fluorescence microscopy [23, 25–28]. Furthermore, the imaging technique has been used to investigate dynamic processes on varying scales, including tracking microtubules plus tips of the mitotic apparatus [29], lineage tracing of cells in spheroids [24] up to *in toto* imaging of mouse cells from zygote to blastocyst [30] as well as whole embryos of *Drosophila melanogaster* [31] and zebrafish [32], further advancing knowledge about developmental processes. These works all involved automated tracking algorithms.

Here, we have adapted the re-aggregation assay, creating embryonic renal organoids rather than flat tissues. This is the first time that re-aggregated kidney rudiments have been imaged by light sheet fluorescence microscopy. Performing long-term time lapse imaging, we aimed to track labelled cells during the culture period, and subsequently analyse the tracking data.

## Materials and methods

### Materials

Materials were purchased from Sigma-Aldrich (Sigma-Aldrich Corp., USA) unless otherwise indicated.

### PDMS-polycarbonate membrane well fabrication

A custom organoid well construct was fabricated using the transparent, biocompatible polymer PDMS (Sylgard 184, Dow Corning, USA) [33]. Briefly, the PDMS pre-polymer mixed with its curing agent (10:1 by weight) was degassed, poured into 35mm Petri dishes and cured at 60°C on a hotplate overnight to ensure full crosslinking. 16 wells were punched into each PDMS disc using a 4 mm disposable biopsy punch (Kai Medical, Inc., USA). The prepared PDMS discs were then autoclaved. Track-etched polycarbonate filters (25 mm diameter, 1.2 μm pores, RTTP02500, Millipore, Watford, UK) were attached in sterile conditions to the bottom of the PDMS disc, covering the 16 punched wells for the culture of the organoids. The polycarbonate membrane and PDMS are both hydrophobic, sealing reversibly yet sufficiently through van der Waals forces. Immediately prior to use, the PDMS organoid culture discs were placed on PDMS separators to create a 2 mm void underneath the polycarbonate membrane in a 6 well plate (Corning) and the respective well was filled with 3 ml MEM (+10% FCS, without phenol red, Fisher Scientific UK Ltd. 11504506). The individual organoid wells were also filled with medium.

### Isolation of embryonic kidneys

For all experiments, kidneys were isolated from E13.5 embryos after humane sacrifice of mice. For the initial experiments, wild-type pregnant CD1 mice (Charles River, Margate, UK) were used. Alternatively, Wt1^+/GFP^ (Wt1^tm1Nhsn^, following Wt1-GFP) [34] male mice were crossed to wild-type CD1 female mice. Experimental animal protocols were performed in accordance with the approved guidelines under a licence granted under the Animals (Scientific Procedures) Act 1986 and approved by the University of Liverpool Animal Ethics Committee. Experimental animal protocols were performed in accordance with the approved guidelines under project licence PPL 70/8741 granted by the Home Office under the Animals (Scientific Procedures) Act 1986 and approved by the University of Liverpool Institutional Care and Use Committee. Intact rudiments were either fixed immediately after isolation or cultured on a polycarbonate membrane (RTTP02500) placed on a metal grid and grown in DMEM (+10% FCS, D5796).

### Re-aggregation assay and organoid culture

The embryonic rudiments were processed using an established protocol [5]. Briefly, they were pooled, washed 3 times with PBS and dissociated in 3 ml of 1× trypsin in PBS at 37°C for five minutes. Every two minutes, the fragments of the rudiments were gently pipetted up and down to assure complete cellular dissociation of the tissue. The fragments/cells were stabilised with 10 ml DMEM + 10% FCS at 37°C for five minutes and centrifuged to obtain a pellet. The re-suspended pellet was then counted by Trypan blue exclusion using a TC20 automated cell counter (Bio-Rad Laboratories, Inc., USA), presenting with an average cell viability of 77% (wild-type) and 69% (Wt1-GFP). For each assay, 1×10^5^ cells were dispensed in 500 μl microfuge tubes and centrifuged at 3000 rpm for two minutes. Each pellet was carefully detached from the tube wall and placed into a PDMS organoid well where the organoids compacted overnight. The organoids were cultured in this setup in a humidified incubator at 37°C and 5% CO_2_ for up to six days (see Table 1 for details on treatment times).

**Table 1:** Time regimes of mouse embryonic kidney organoid and organ treatments.

Time (d)	Endpoint fixing and staining		Functional assay		Live imaging	
	Organoid	Kidney	Organoid	Kidney	PNA stain pilot	Wt1-GFP
**0**	Re-aggregation	Fix	Re-aggregation	Culture	Re-aggregation	Re-aggregation
**1**	Culture		Culture	Culture	Culture	Embedding of spheroids in agarose-gelatine hydrogel and live imaging
**2**	Culture		Culture	Culture	Culture	
**3**	Culture		Culture	Culture	Culture	
**4**	Culture		Culture	Functional assay	Embedding of spheroids in agarose-gelatine hydrogel and live imaging	
**5**	Culture		Culture			
**6**	Fix		Functional assay			
	Acrylamide hydrogel embedding for clearing	Acrylamide hydrogel embedding for clearing				

### Gel embedded live culture of organoids

Live imaging of organoids was performed on samples that were embedded in a hydrogel cylinder consisting of an agarose-gelatine mix inside a glass capillary. The hydrogels were prepared in sterile conditions. Prior to use, agarose aliquots consisting of low melting agarose (3% in PBS, Sigma A9414) were melted in a heat block at 75°C before cooling down to 38°C. Gelatine aliquots (6% in PBS, Sigma G1890) were heated to 38°C and the two hydrogels were mixed 50:50 v/v before embedding the organoids. The appropriate capillary sizes (size 1 ~0.68 mm, size 2 ~1 mm, size 3 ~1.5 mm, size 4 ~2.15 mm) were chosen for the sample to occupy between 1/3 and 2/3 of the agarose diameter. For culturing and imaging, the hydrogel cylinder was partially extruded following solidification so that the organoids were located outside the capillary. Spheroids for live imaging were pre-stained in phenol-red free MEM (10% FCS, 1% penicillin/streptomycin) containing 30 μg/ml rhodamine-labelled Peanut agglutinin (PNA, Vector Laboratories, USA) for 30 mins in an incubator at 37°C with 5% CO_2_. The microscope imaging chamber was sterilised with 70% ethanol and rinsed with ddH2), sterile PBS and subsequently medium before filling it with ~12ml of phenol red-free MEM supplemented with 10% FCB, 1% penicillin/streptomycin and 3μg/ml PNA-rh.

### Fixing

Medium was removed from the incubated samples and intact kidneys, pellets and organoids were washed with PBS followed by fixation with 4% paraformaldehyde (PFA) for 30 mins at room temperature and three further PBS washes.

### Clearing

The clearing protocol chosen for this study is based on the CLARITY method [35–38], involving the embedding of the tissue in an acrylamide-based hydrogel followed by the removal of the main scattering components, i.e. lipids. The removal of the lipids also enables a deeper penetration of immunostaining agents, ultimately resulting in optically cleared tissue whilst maintaining structural information.

#### Hydrogel preparation

The embedding hydrogel was prepared according to an established protocol [36]. The materials used to make up 40 ml of the final monomer solution are the following: 4.7 ml of Acrylamide/Bis (30%, 37.5:1), 100.5mg of VA-044 Initiator (2,2'-Azobis[2-(2-imidazolin-2-yl)propane]dihydrochloride, Alphalabs 017–19362, final concentration 0.25%), 4 ml of 10× PBS (final concentration 1×) and 10 ml of 16% PFA (final concentration 4%) in 21 ml ddH_2_O. The individual components were kept on ice during mixing to avoid polymerisation. The final solution was stored at -20°C until needed.

#### Hydrogel sample embedding

Hydrogel monomer aliquots were thawed at 4°C or on ice and then gently mixed to disperse any precipitate. The sample was introduced into the hydrogel monomer solution and kept on ice. They were incubated on a rocker overnight at 4°C for hydrogel infusion. Prior to polymerisation, a layer of peanut oil was added on top of the aliquots and polymerisation was initiated and completed by keeping the samples at 37°C for 3 hours in a heating block. Following polymerisation, the peanut oil was discarded and excess hydrogel was removed from the samples using dissection tools, followed by washing 3× with PBS and storage at 4°C until needed.

#### Lipid removal

The final clearing solution was prepared by dissolving 3.2 g of SDS in 40 ml of ddH_2_O under agitation and stored at room temperature. The samples were cleared through passive clearing [38] by immersing the samples in the clearing solution and incubating them at 37°C for 48 hours. Finally, the samples were washed 3× with PBST (0.1% Tween in PBS) for 10 mins to remove remaining SDS micelles. The cleared samples were stored in PBST at 4°C until needed.

### Immunofluorescence of fixed organoids

Samples, some cleared and some un-cleared, were blocked for 2 hours at room temperature in blocking buffer (10% goat serum with 0.1% TritonX in PBS). The samples were incubated with primary antibodies diluted in blocking buffer at 4°C using conditions shown in Table 2. Secondary antibodies were diluted at a concentration of 1:1000 in blocking buffer and incubated for 2 hours at room temperature prior to staining with 30 μg/ml PNA for 30 mins. The secondary antibodies used were: Alexa Fluor^™^ 405 Goat Anti-Rabbit IgG (H+L), Alexa Fluor^™^ 633 Goat Anti-Mouse IgG_1_, Alexa Fluor^™^ 488 Goat Anti-mouse (Invitrogen Fisher Scientific).

**Table 2:** List of primary antibodies and stains used.

Renal structure	Antibody	Supplier	Product	Dilution	Incubation time
Basement membrane	**Laminin**	Sigma-Aldrich	L9393	1:1000	Overnight
Tubular lumen	**Megalin**	Acris	DM3613P	1:200	Overnight
Ureteric bud incl. tips	**Cytokeratin**	Abcam	Ab115959	1:100	Overnight
Metanephric mesenchyme	**Pax2**	Abcam	Ab37129	1:200	Overnight
Metanephric mesenchyme	**Six2**	Proteintech	11562-1-AP	1:200	Overnight
Podocytes in glomeruli, Metanephric mesenchyme	**Wt1**	Millipore	05–753	1:200	Overnight
Renal podocytes	**Synaptopodin**	Progen	65194	1:1	Overnight
Renal podocytes	**Nephrin**	Abcam	Ab72908	1:180	Overnight
Basement membrane	**Rhodamine labelled Peanut Agglutinin (PNA)**	Vector Laboratories	RL-1072	20–30 μg/ml	30 mins

### Functional (organic anion transport) assay

All assays were performed on live cultures. The intact kidney rudiments were cultured at the air liquid interface for 4 days following dissection. The organoids were cultured under medium immersion for 6 days following dissection. The samples cultured at the air liquid interface were gently removed from the membranes before starting the assay.

The samples were incubated for 1 hour at 25°C with PBS containing 1μM 5(6)-carboxyfluorescein (6-CF) and 20 μg/ml of PNA (Vector Laboratories). For the control samples, 2 mM probenecid was also added to the solution to inhibit the organic anion transport. After incubation, the samples were washed with ice cold PBS for 10 mins followed by an incubation with 8mM probenecid in PBS for 15 mins to arrest any transport. The functional assay was concluded by two PBS washes and samples were then embedded in 1.5% agarose for imaging.

### Imaging

Fixed and stained samples were embedded in 1.5% agarose columns using appropriately sized glass capillaries. The glass capillaries were inserted into the imaging chamber of the microscope and the agarose column was extruded into the chamber filled with PBS. The light sheet fluorescence microscope used was a Zeiss Lightsheet Z.1 microscope, fitted with 10× illumination objectives and an achromatic 20× detection objective. The laser wavelengths used were 405 nm, 488 nm, 561 nm, 638 nm and the laser intensity was kept to a minimum. Green fluorescent beads, 30 nm small beads (polystyrene latex beads (470/505), Sigma-Aldrich Corp., USA) or 1 μm large beads (Fluospheres (505/515), Thermo Fisher Scientific Inc., USA), were used as fiduciary markers.

### Data analysis

Data analysis was performed using the Zen analysis software (Carl Zeiss AG, Germany) as well as Fiji (ImageJ) [39]. Cell tracking was performed using the TrackMate plugin (v.2.8.2) for Fiji [40] and three-dimensional visualisations of the data were generated using the 3D Viewer (v.) plugin for Fiji [41]. The TrackMate [42] plugin for Fiji is a single particle tracking tool that identifies spots, i.e. cells, in every frame and the trajectories of cells are reconstructed by assigning an identity over these frames in the shape of a track. The positional data and numerical features generated using TrackMate were further processed and displayed using Matlab (R2014a, The Mathworks Inc., USA). Violin plots were generated using a script obtained via https://github.com/bastibe/Violinplot-Matlab. The Mann-Whitney U procedure for statistical testing was run in Matlab. Multiview reconstruction was performed using the MultiView Reconstruction plugin in Fiji [43, 44]. Object segmentation and volume calculation of the segments was done in Imaris (8.4.2, Bitplane AG, Switzerland).

## Results and discussion

**Fig 1:**
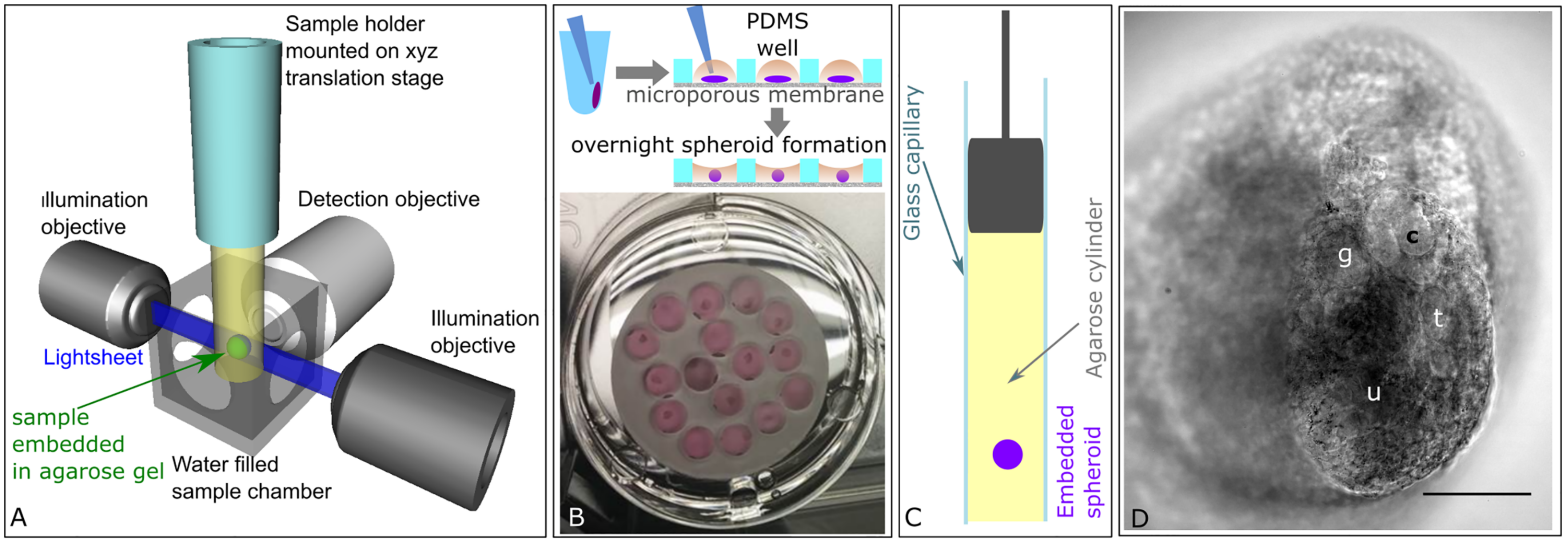
Experimental setup. *A) Light sheet fluorescence microscopy*. A single plane of the specimen, embedded in an agarose gel cylinder, is illuminated by a light sheet with the detection located orthogonally to the illumination. Only the plane that is observed is illuminated, resulting in vastly reduced photo bleaching and phototoxic effects. The specimen can be moved and rotated in the fluid filled chamber and through the stationary light sheet. *B) Embryonic kidney organoid assay*. To generate organoids, the renal pellets were removed from the centrifuge tubes and placed in organoid wells in the PDMS disc. Overnight, the pellets compacted into organoids. C) For light sheet imaging, the organoids were embedded into an agarose cylinder inside a glass capillary. D) Transmitted light image of a renal tissue spheroid embedded in agarose. The focal plane shows several renal structures including a tubule (t), ureteric bud tip (u), comma shaped body (c) and glomerulus (g). Scale bar = 100 μm.

We have adapted the kidney rudiment re-aggregation assay by creating embryonic renal organoids suitable for light sheet fluorescence microscopy (Fig 1). These organoids and also intact kidney rudiments were imaged with a light sheet fluorescence microscope after end point fixing and staining to determine the organotypical development of structures in the organoids. Furthermore, we aimed to image the tissues *in vitro* over the long-term to track labelled cells during the culture period, and subsequently analyse the tracking data.

### Optimising protocols for generation of re-aggregated embryonic renal organoids for light sheet microscopy

For post-fixation imaging of the developing kidney structures, we generated re-aggregated organoids from embryonic kidneys at E13.5 following previously published protocols [5, 6, 45, 46]. The pellet was transferred into an organoid well fabricated in a PDMS disc, where the size of the well and its surface hydrophobicity promoted pellet compaction overnight, leading to the formation of an organoid that did not attach to the well (Fig 1B). The micro-porous membrane at the bottom ensured the exposure of the organoid to growth factors in the medium, thus supporting its growth and enabling the recapitulation of developing structures (Fig 1D). For live imaging in a light sheet fluorescence microscope, we embedded the samples in an agarose-gelatine hydrogel using glass capillaries and cultured them for up to 15 hours while recording (Fig 1C and 1D). The agarose-gelatine hydrogel mixture supported the development of nephron structures and the addition of a fluorescent vital stain. Turning the gel column around the gravitational axis, we could repeatedly image the organoids from various angles.

Throughout the 6 day culture period, we observed a reduction in size of the organoids, both in those cultured in medium and in those cultured in the agarose-gelatine hydrogel (Fig 1D and S1 Fig). On day 4, the organoids cultured in an agarose-gelatine gel shrank to ~57% of the size recorded 24 hours after re-aggregation, i.e. after organoid formation (n = 3). As the renal cells do not proliferate indefinitely, an increase in organoid size is not expected. The reduction in size probably occurs in two stages. Generally, in organoid models, a reduction in organoid size is observed during tissue coalescence. Furthermore, Levefre et al. have found that the cap mesenchyme population in re-aggregated kidney rudiments declines steadily over a culture period of up to 48 hours [47], which was attributed to cell death. Furthermore, we did not use ROCKi in the culture that was used to improve cell survival in previously reported protocols using E11.5 kidney rudiments [5] and considered unnecessary by Lefevre et al.

### Renal marker analysis in fixed embryonic kidneys and re-aggregated renal organoids

To demonstrate that the culture method above resulted in 3D kidney organoids with appropriate organotypic structures, we probed PDMS-cultured organoids with a panel of markers (Table 1). As a comparison and control of the specificity of the markers, we included intact embryonic kidney rudiments (E13.5). Maximum intensity projections of stained embryonic kidneys and organoids allowed a quick comparison of their three-dimensional arrangement since maximum intensity projections plot the signal of the respective markers distributed throughout the samples onto one plane.

Light sheet fluorescence microscopy imaging of laminin-stained embryonic kidneys and organoids showed an arrangement of basement membranes which could indicate both the development of the ureteric bud and/or tubules (S2 Fig). The proximal tubule-specific receptor megalin was rarely detectable in the embryonic kidneys at E13.5 [48] (Figure B in S2 Fig), i.e. laminin^+^ structures probably indicate ureteric bud and immature proximal tubules. In the more mature organoids, megalin was expressed within the lumen of the majority of laminin^+^ structures (Figures C and D in S2 Fig), consistent with the development of epithelial tubules [5]. Cytokeratin-stained embryonic kidneys revealed the presence of collecting duct trees with several branches ending in ureteric buds (Figures A and E in S3 Fig) [49]. Similarly, branched though less organised cytokeratin^+^ structures could be identified in 6-day old organoids (Figures C and G S3 Fig).

We included the ureteric bud in the generation of the single cell suspension. As a result, the ureteric bud cells formed independent epithelial foci which did not lead to the formation of a sole ureteric tree. To circumvent this problem, alternative re-aggregation protocols start from the separation of the metanephric mesenchyme from the ureteric bud, where the ureteric bud is either discarded or cultured separately and then re-added to the metanephric mesenchyme mix at the beginning of the culture period [2, 50]. This method results in a more physiological renal tissue aggregated around one ureteric bud tree.

For the detection of cap mesenchyme we used Pax2 antibody staining, which was arranged around basement membrane-bound ureteric bud structures in intact kidneys and re-aggregated organoids (S4 Fig). Pax2 was highly expressed in the condensing mesenchyme but was also detectable within the cells of the tubules (Figures E and G in S4 Fig) [51]. In both the intact embryonic kidneys and organoids, the cap mesenchyme consisted of 4–5 cell layers (Figure E-H in S4 Fig, S1 Video). Re-aggregated organoids also expressed the nephron progenitor marker Six2 in the cap mesenchyme (S5 Fig).

Light sheet fluorescence microscopy has the advantage to image much deeper into the tissue than traditional confocal microscopy. However, the penetration of antibodies was limited and this was particularly the case for the laminin antibody. To exploit the deep imaging capability of the light sheet microscope, the pan-epithelial lectin stain PNA was used as an alternative marker for basement membranes. The stain only required one rapid staining step, penetrated fully through the samples, and was therefore used throughout this study to highlight the networks of ureteric bud branches and tubules within embryonic kidneys and organoids (S3 and S4 Figs). Figure B in S5 Fig shows a panel of single focal planes of a stained organoid at increasing depth. Only the endogenous Wt1-GFP and the PNA signals were detected throughout the spheroid, while the antibody signals (Six2, Synaptopodin) were limited to 75 μm from the organoid surface.

### Detecting glomerular maturation in spheroidal re-aggregated kidney organoids

**Fig 2:**
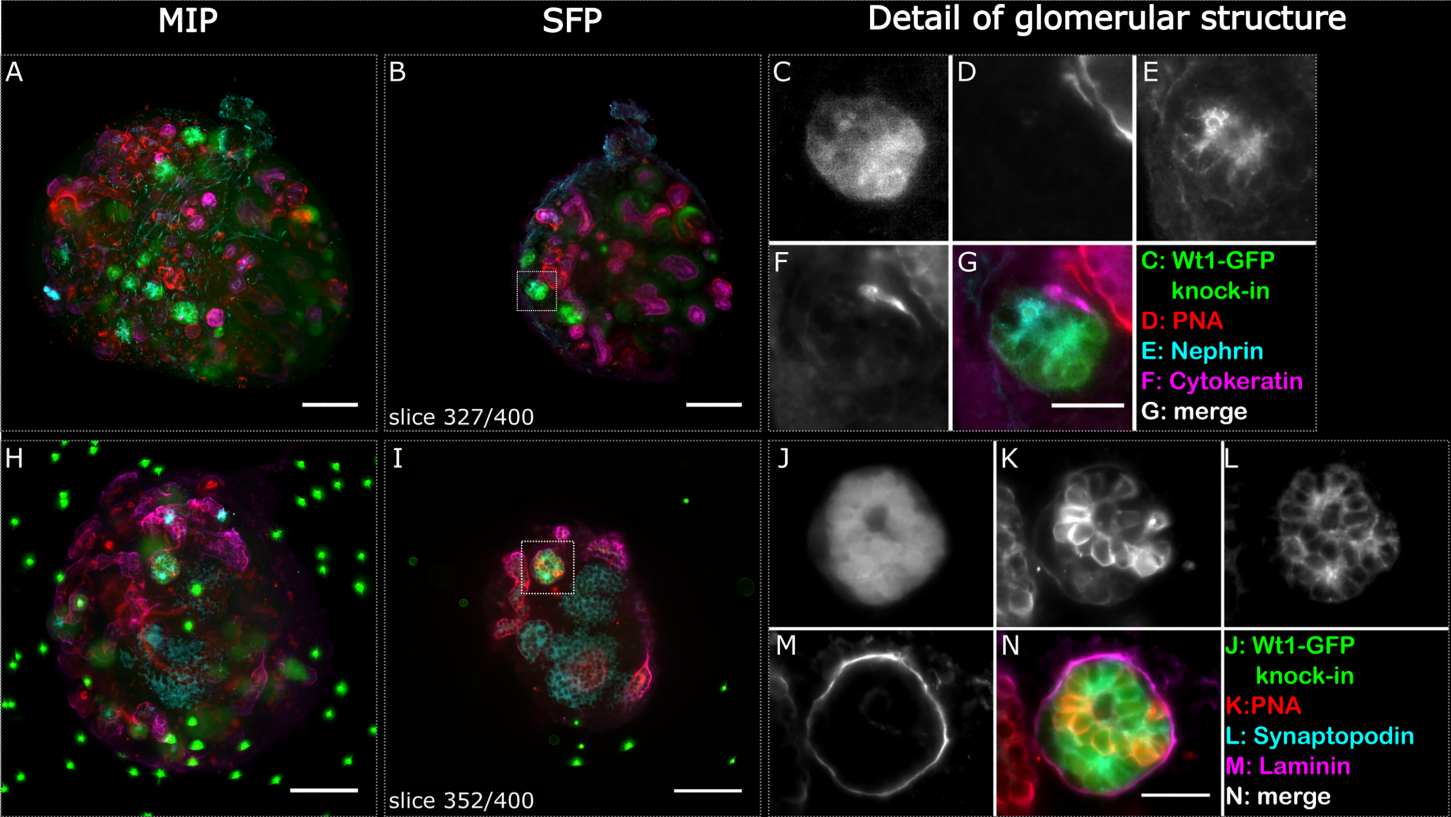
Glomerular structures stained positively for podocyte markers indicating phenotypic maturity in re-aggregated embryonic renal organoids after 6 days of culture. Two whole organoids (A-G and H-N) were imaged in the light sheet microscope and are depicted as maximum intensity projections (A, H) and respective selected single focal planes (B, I). Glomerular structures are highlighted with a dashed square in the single focal plane, and detailed images are shown in C-G and J-N respectively. The Wt1-GFP structure in C also stained positive for nephrin (E), and the Wt1-GFP structure in J stained positive for synaptopodin (L). Cytokeratin highlights the various ureteric bud foci, and PNA and laminin the basement membranes of the ureteric bud and nephric tubules. The sample shown in H-N contains fluorescent beads as reference points for multi view reconstruction of the dataset. The organoids were not cleared. Scale bars in A, B, H and I: 100 μm, G: 10 μm, N: 25μm.

To track developmental cell changes in renal structures over time in the light sheet microscope, we used embryonic kidneys from Wt1-GFP knock-in reporter mice where GFP is expressed under control of the endogenous Wt1 promoter. Wt1 is dynamically expressed throughout kidney development in the metanephric mesenchyme, cap mesenchyme, renal vesicles, S- and comma-shaped bodies and podocytes [52–55]. We found that Wt1 was expressed in the cap mesenchyme, renal vesicles, S and comma-shaped bodies in both intact, fixed E13.5 kidneys and organoids cultured in the PDMS-polycarbonate membrane well for 6 days (S6 Fig). Due to the difference in the developmental stages of the embryonic kidneys and organoids [54, 55], we only observed a strong signal in the maturing podocytes of the glomeruli in the organoids. In maximum intensity projection and single focal plane images of two organoids, fixed and stained after 6 days of culture, several bright Wt1+ structures were visible that co-labelled with the podocyte markers nephrin or synaptopodin, respectively (Fig 2A and 2H).

Nephrin (Fig 2E) is a slit diaphragm protein and part of the renal filtration barrier essential for maintaining normal glomerular permeability. In kidney organoids cultured for 6 days, the marker is present on the apical surface of Wt1-expressing podocytes (Fig 2C). In another staining panel, the Wt1+ clusters (Fig 2J) co-labelled with synaptopodin (Fig 2L). The expression of synaptopodin in podocyte foot processes is differentiation-dependent, indicating advanced cytoskeleton development and is considered an important marker of the phenotypic maturity of podocytes. We stained for synaptopodin and nephrin in E13.5 kidney rudiments but did not see any strong labelling. According to in situ hybridisation images deposited in the GUDMAP database [54, 55], a positive stain for synaptopodin should be expected at E15 and for nephrin at E18. These results indicate normal organotypic development of glomeruli in the re-aggregated kidney organoids [56] despite a lack of a well-integrated and connected ureteric tree. In that respect the spheroids resemble re-aggregates cultured at air-liquid-interface. Re-aggregates cultured at air-liquid interface have been used previously to determine the fate of undifferentiated and differentiated embryonic stem cells as well as kidney derived stem cells [6, 7] and therefore we proceeded with the spheroid setup.

### Physiological tubular transport in re-aggregated kidney organoids

**Fig 3:**
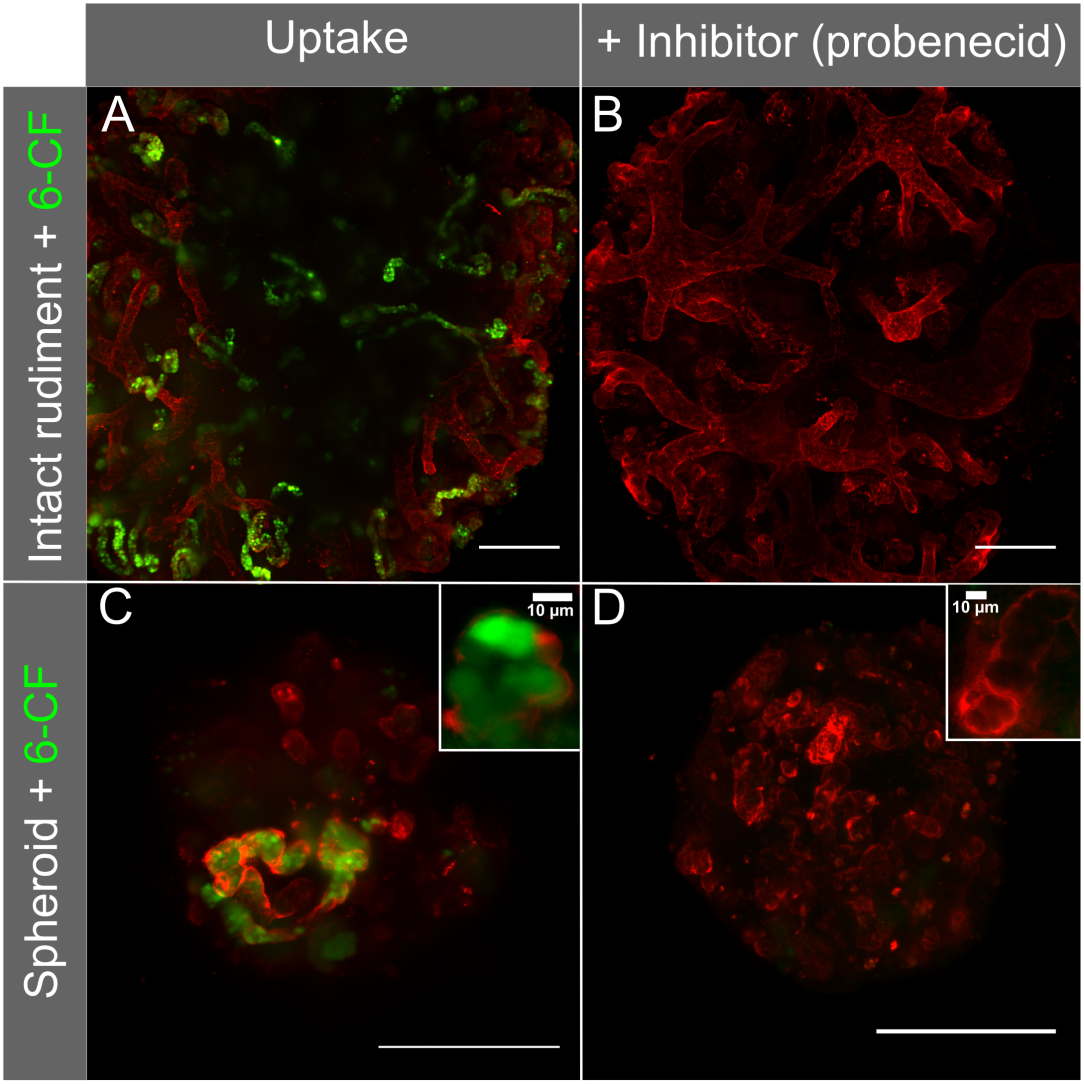
Specific uptake of the fluorescent anion 6-Carboxyfluorescein with and without the organic anion transport inhibitor probenecid. The basement membranes of the tubules in the cultures were highlighted with rhodamine-conjugated PNA (red). Uptake was investigated in E13.5 mouse rudiments cultured for 4 days on a micro-porous membrane at air-liquid interface, and re-aggregated organoids cultured for 6 days immersed in a PDMS well system with a bottom layer of micro-porous membrane. The images are maximum intensity projections of z-stacks. Scale bar = 200μm.

It has been shown previously that serially re-aggregated kidney rudiments develop the tubular transport function from three days in culture whereas transport function in cultured intact kidneys appeared as early as two days in culture [57]. To test the physiological function of the developed tubules in 6-day old spheroidal re-aggregated kidney organoids, we employed the same organic anion transporter assay. Specifically, we assessed whether the tubule cells were capable of organic anion transporter-dependent uptake of the organic anion mimic 6-Carboxyfluorescein (6-CF). For comparison, we included intact embryonic kidney rudiments collected at E13.5 that had been cultured for 4 days at the air-liquid interface. Since organoids lag behind in their developmental progress due to the re-aggregation process, the intact rudiments were cultured for 4 days to reach a maturity similar to 6-day old organoids. Our analysis showed that in both the cultured embryonic kidneys and the re-aggregated organoids, 6-CF was transported into the cells (Fig 3A and 3C). Furthermore, the organic anion transporter-based 6-CF uptake could be successfully prevented using the inhibitor probenecid (Fig 3B and 3D), both in organoids and cultured embryonic kidneys. These findings demonstrate active organic anion transport in renal tubules formed in the spheroid re-aggregated embryonic kidney organoids and confirm previous results in intact rudiments [6]. Our results agree with previous organic anion transporter assays that have shown that tubules, developed in re-aggregated engineered kidney tissue, exhibit transport function, thus mimicking physiological processes [6, 57].

### Live imaging and cell tracking in spheroidal organoids cultured inside agarose-gelatine hydrogel

We could demonstrate that organotypic structures can form in embryonic kidney organoids after re-aggregation. Aiming to record cell migration and differentiation we imaged the organoids live, by culturing them inside hydrogel cylinders surrounded by medium within the light sheet fluorescence microscope. In a pilot experiment, we generated wild-type embryonic mouse kidney organoids followed by 4 day culture in the PDMS wells, before embedding in the hydrogel cylinders. We chose a 4-day old organoid because based on our previous results we expected that ureteric bud foci and nephric tubules had been established and their basement membranes should stain readily, providing structural context for the spheroid. The sample chamber was filled with medium containing a low concentration of PNA, for continuous staining during live cell imaging. The compaction of the organoid over time was clearly visible (Figure A in S7 Fig). The dye gradually diffused into the tissue. Initially, the PNA signal seemed indiscriminate and no structures were recognisable up until 4 hours into time series (S2 Video). Subsequently, live samples were pre-stained prior to imaging to avoid the delay in structure detection. Throughout the time series individual, labelled cells could be identified and tracked, including cell integration into a tubule (Figure B in S7 Fig, S2 Video). The development of renal structures in 4-day old organoids can therefore be visualised over a 13 hour time period in light sheet live cell imaging.

**Fig 4:**
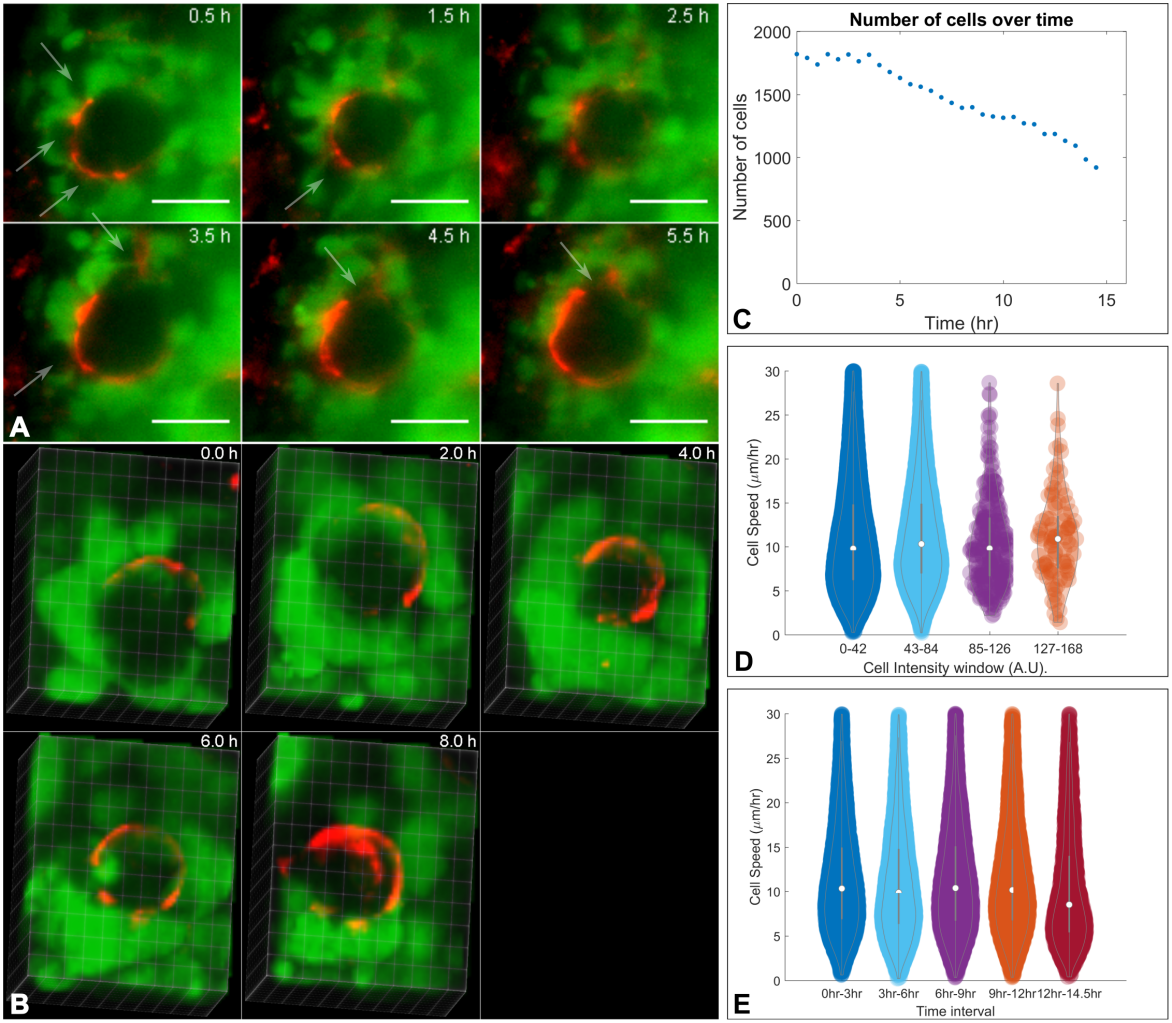
Live imaging of renal organoids using light sheet fluorescence microscopy. A) Detailed views of representative single focal plane images showing Wt1-GFP cells (green) grouped around a ureteric bud structure labelled with PNA (red). Over the depicted period of 5 hours, Wt1-GFP cells were entirely absent from the ureteric bud. Most of the cells surrounding the ureteric bud structure expressed GFP but there were also some dark cells (arrows). Scale bar 25 μm. B) shows a three-dimensional render of another basement membrane surrounded structure void of Wt1-GFP cells within the same organoid over a time period of 8 hours. C-E show a selection of parameters analysed following automated cell tracking. (C) The overall number of GFP expressing cells declined slowly over time. (D) Cells were grouped into different intensity windows to compare the speed at which they moved, revealing no notable difference. The cell intensity distribution is skewed towards low intensities. (E) The cell speed does not change much over the 15 hour imaging period.

**Fig 5:**
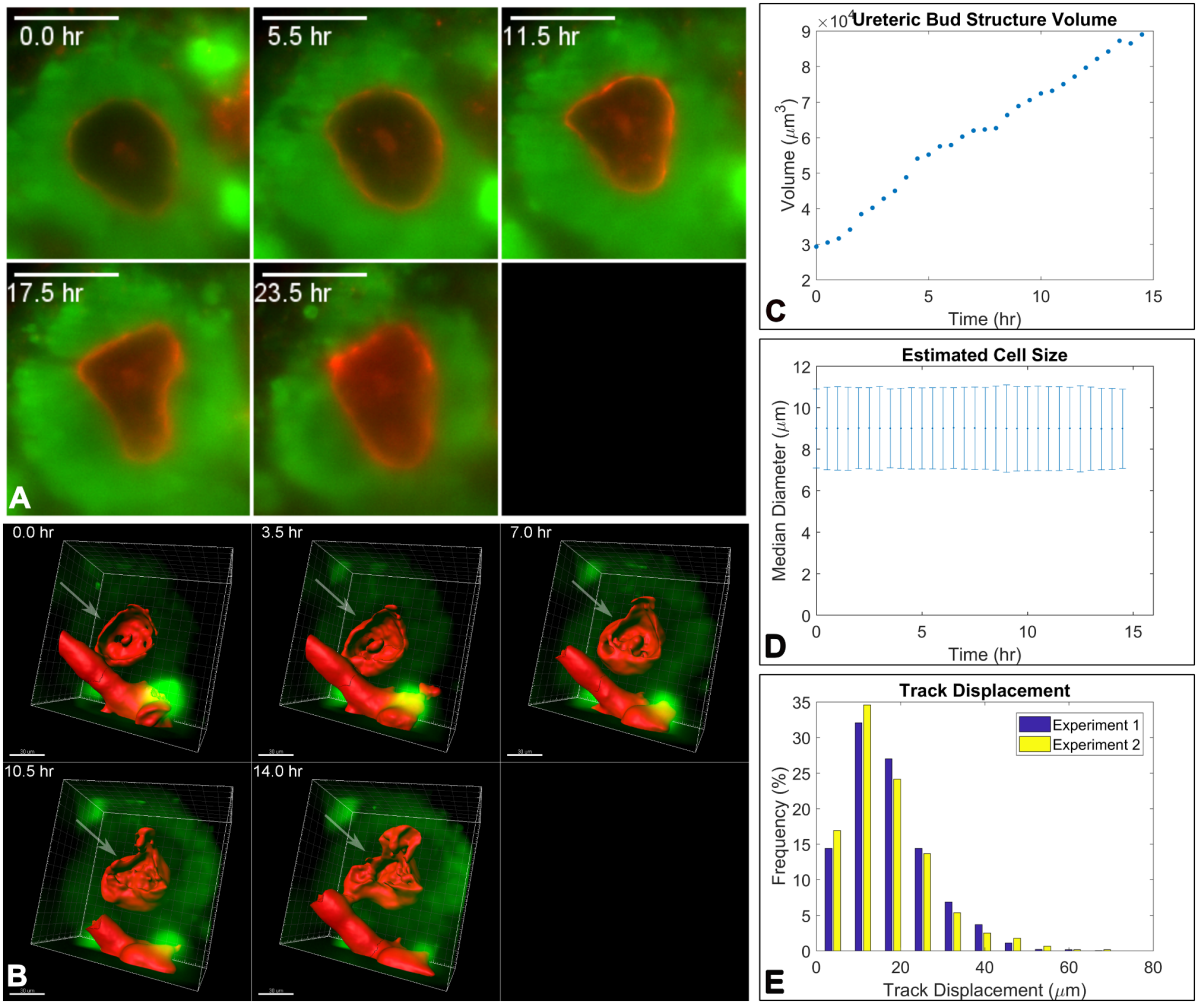
Live imaging of renal organoids using light sheet fluorescence microscopy. A) Detailed views of representative single focal plane images showing Wt1-GFP cells (green) grouped around a ureteric bud structure labelled with PNA (red). Over the depicted period of 23.5 hours, Wt1-GFP cells were entirely absent from the ureteric bud. Every cell surrounding the ureteric bud structure appeared to express GFP. Scale bar 25 μm. B) Three-dimensional segmentation of the ureteric bud structure shown in A). The ureteric bud segment is indicated with an arrow. The rod-shaped segment at the bottom is a piece of dust integrated in the organoid. Scale bar: 30 μm. C) represents the volume increase of the ureteric bud structure throughout the time series. D) shows that the cell diameter does not change over time. E) shows a comparison of the track displacement histograms of the two time series discussed in this and the previous figure, which are similar.

To investigate the cell dynamics during nephron formation in live organoids, we cultured re-aggregated renal organoids from Wt1-GFP reporter mice in PDMS dishes for 24 hours (Table 1), pre-stained with PNA, and then embedded within the hydrogel cylinders for continued culture and live imaging. During the imaging period and while structures developed, the Wt1-GFP cells continually expressed GFP, allowing the automated tracking of cells over time (S3 and S4 Videos). Typically, PNA-labelled structures were visible at the start of the imaging series, i.e. 24 hrs following cell re-aggregation (Fig 4A for “Experiment 1”, T = 0.5 hr and Fig 5A, T = 0 hr, and S5 Video for “Experiment 2”). For both experiments, Wt1-GFP cells were grouped around PNA-labelled basement membrane bound structures whereas the inner parts of the structures were entirely devoid of Wt1-GFP cells throughout the imaging periods of 15 hours. This pattern reflects the situation *in vivo*, where the ureteric bud in mouse embryonic kidneys is surrounded by Wt1^+^ cap mesenchyme cells. Most of the cells surrounding the ureteric bud in Fig 4A expressed GFP but some did not (arrows). As the cells are not labelled with any other marker, it is unclear whether they started expressing GFP at later time points or moved away from the cap mesenchyme. Fig 4B shows a three-dimensional render of another area within the same organoid, containing a second basement membrane bound structure void of GFP expressing cells but surrounded by them over a period of eight hours.

As we captured the three-dimensional arrangement of living GFP-expressing cells within the organoids every 30 minutes for 15 hours with the light sheet microscope, we could track their positions over time. The single particle tracking tool TrackMate identifies cells and their tracks throughout the time series, generating a range of data sets. We plotted the cell distribution in three-dimensional space and calculated the median distance of each cell to the centre of mass of the organoid. This enabled us to estimate the size of the organoid and its change over time, thus quantifying compaction (S6 Video). Some of the quantitative data derived from Experiment 1 is represented in Fig 4C–4E. The number of Wt1-GFP cells declined over time by 50% at 14 hrs (Fig 4C), giving some indication that de-differentiation and/or cell death occur. The median cell intensity also declined during the time series (S8 Fig) and viewing the time series in detail shows that there is cell death of Wt1-GFP expressing cells. Quantitative parameters like the cell speed and median intensity can be correlated at the single cell level (Fig 4D). Parameters like the track median speed can be plotted as a histogram over the whole imaging period but more information can be derived from windowing the speed of cells (Fig 4E). The violin plot shows that the cell speed distribution remains constant throughout the first 12 hours followed by a slight drop during the 12–14.5 hr interval. After >20 hours of culture, an infection was apparent in the surrounding medium and the experiment had to be terminated. We are not able to differentiate cell death due to factors internal or external of the spheroid.

To evaluate reproducibility we repeated the time series experiment, setting Experiment 2 up identically to Experiment 1. Detailed views of a single focal plane of Experiment 2 over 23.5 hours are shown in Fig 5A. As in Experiment 1, a basement membrane-bound structure surrounded by Wt1-GFP expressing cells is clearly identifiable. Fig 5B shows a 3D segmentation version of the same structure (arrow). The volume was calculated from the segmentation and increased steadily (Fig 5C). The shape of the ureteric bud changed over time, ultimately resembling two tips emanating from the original spherical shape. The mean cell diameter remained constant over time (Fig 5D) but the number of cells decreased like in Experiment 1.

Quantitative tracking data enables quantitative comparison between experiments, which was not possible previously. The mean cell diameter of Wt1-GFP cells determined via TrackMate is similar for both time series (9.7 μm and 9.0 μm). Fig 5E shows the track displacement histograms of both experiments that were set up identically on different days. The distributions have a similar shape with the mean of the track displacement of 15.4 μm (n = 3026) for Experiment 1 and 14.2 μm (n = 1423) for Experiment 2. Therefore, obtaining quantitative data through live imaging in the light sheet fluorescence microscope, of the developmental processes that can be tracked, opens up an avenue to statistical analysis.

## Conclusion

We show that we can reliably create renal tissue organoids from disaggregated mouse embryonic kidneys using a custom construct made of PDMS. We created a customizable multi-well enabling the formation of up to 16 organoids in parallel, hence reducing the associated costs and intra-experimental variability. This set-up also holds potential to create higher-throughput platforms for drug testing and additional studies. The generated renal and chimeric tissue organoids were imaged with light sheet fluorescence microscopy. Structure and tubular function were preserved *in vitro*. Within the organoids imaged cells long-term, and subsequently tracked the cell fate and generated previously unavailable quantitative cell data.

## Supporting information

S1 Fig Compaction. A) Compaction and increasing optical density of a pellet cultured at air-liquid interface. The pellet was incubated on a micro-porous membrane and the label in the top left corner indicates the day of culture. At day two and three, the pellet has a comparatively large footprint (white outline) on the membrane whereas at day five, the cells have self assembled into a tissue with a much smaller footprint that appears much darker with some visible tubule-like structures. B) Organoid compaction over time measured as the equatorial area of organoids over five days. The agarose + gelatine refers to organoids that were embedded into this gel mixture 24 hours after re-aggregation. Error bars are the standard deviation of the normalised data.
(TIF)

S2 Fig Immunostaining for tubular lumen with megalin and tubule and ureteric basement membranes with laminin. (A) shows the maximum intensity projection and (B) a single focal plane of cleared whole mount fixed and stained mouse embryonic E13.5 kidneys. There are few megalin^+^ structures in the whole rudiments (arrows), representative of the early developmental stage of the kidneys where only few tubules have developed. (B) shows large laminin^+^ structures which are megalin^-^, indicative of the ureteric bud. (C) shows the maximum intensity projection and (D) a single focal plane of a cleared organoid that was fixed after six days of culture. There are many megalin^+^ structures, surrounded by laminin^+^ membranes, indicative of nephric tubules (arrows). For both, kidney rudiments and specifically organoids, the megalin penetration was superior to the laminin penetration and there are megalin^+^ structures that appear to be laminin^-^, which we consider untrue. Increasing the incubation times resulted in increased unspecific staining but not in improved penetration depth. Had the laminin stain penetrated deeper, we would have expected staining around those megalin^+^ lumens as well. All samples were cleared. Scale bars 100 μm.
(TIF)

S3 Fig Immunostaining for the ureteric bud marker cytokeratin and basement membrane staining (PNA). A-D show maximum intensity projections and E-H show single focal plane images of whole mount fixed and stained mouse embryonic E13.5 kidney rudiments and organoids that were cultured for six days before fixing. Both, the cytokeratin and PNA staining reveal branched structures (arrows) in the intact rudiments and the spheroids. In the intact kidney rudiment in (A), the cytokeratin^+^ cells are arranged in the typical tree shape of the ureteric bud, i.e. an intricate branch system connected to one base structure. The tree shape of another embryonic kidney is also highlighted by the basement membrane stain PNA in (B). In the spheroid in (C), the cytokeratin^+^ cells are not organised in an interconnected structure. There are many independent cytokeratin^+^ structures that have developed from different ureteric bud foci, causing a less organised ureteric tree structure, which is also reflected in the PNA staining, which simultaneously highlights developing nephrons. All samples but the spheroid shown in C/G were cleared. Scale bars 100 μm.
(TIF)

S4 Fig Immunostaining for the cap mesenchyme marker Pax2 and basement membrane staining (PNA). A-D show maximum intensity projections and E-H show single focal plane images of whole mount fixed and stained mouse embryonic E13.5 kidneys and organoids that were cultured for six days before fixing and staining. The Pax2^+^ cells are grouped around structures highlighted with PNA, i.e. cap mesenchyme (arrows with dot end). E-H show that the cap mesenchyme consists of 4–5 layers of cells, both in intact kidney rudiments and 6-day old organoids. Pax2+ cells are also present in structures surrounded by basement membrane, indicating ureteric bud (arrows). All samples were cleared. Scale bars 100 μm.
(TIF)

S5 Fig Immunostaining for the cap mesenchyme marker Six2. The images in (A) represent a single focal plane of a 6-day old renal organoid expressing Wt1-GFP and stained with PNA to label basement membranes, Six2 for cap mesenchyme and Synaptopodin for podocytes. The Six2^+^ cells are aligned around ureteric bud highlighted with PNA (arrows with arrow head ends) and also slightly express Wt1-GFP. In the centre of the spheroid, there is an s-shaped body (arrow with dot end), which contains cells expressing Wt1-GFP. A glomerulus (arrow) is located right of the s-shaped body and strongly expresses Wt1 and is also Synaptopodin^+^. This staining pattern indicates that there are mature renal structures and nascent tubules within the spheroid at the same time. (B) shows the same organoid but at different depth indicated in the left top corner of each frame. The PNA and Wt1 signal are strong throughout the whole depth but the Six2 signal is limited to the first 75 μm and unfortunately absent deep in the tissue. This organoid was not cleared. The organoid was recorded from five different angles and the data was successfully fused in Fiji. Scale bars: 100 μm.
(TIF)

S6 Fig Wt1-GFP knock-in expression pattern in intact E13.5 kidneys and organoids cultured for 6 days. The same developmental structures—except glomerulus-like structures—can be identified in the *in vivo* and *in vitro* tissues: metanephric mesenchyme (m), cap mesenchyme (c), renal vesicle (r), glomerular structure (g). The Wt1-GFP signal was weak in the metanephric mesenchyme, increased in the cap-mesenchyme and further increased as the tubular and in particular the glomerular stages are reached. MIP: Maximum Intensity Projection, SFP: Single Focal Plane, Scale bar: 100 μm.
(TIF)

S7 Fig Live imaging of renal organoids using light sheet fluorescence microscopy. (A) maximum intensity projections of an organoid cultured for four days before embedding into a hydrogel cylinder, continued culture in the microscope and imaging (T = 0.0 hr). Over time, the PNA dye penetrated the organoid and tubular structures became visible. The dashed rectangle in the 13 hr panel highlights the area showing as a detailed view of the same time series in B. The movement of a single cell (arrow) migrating towards and integrating into a tubule at T = 8.5 hr can be followed throughout the course of the time series. Scale bar: 25μm.
(TIF)

S8 Fig Cell intensity distributions for five regular time points of the 15-hour time series. The distributions remain similar over time with a slight decrease of the mean in Times Series 1 (A) and a slight increase of the mean in Time Series 2 (B).
(PNG)

S1 Video Cap mesenchyme around developing structures at day 6. Video showing a three-dimensional rendering of an isosurface fitting of basement membrane positive tubules (red) surrounded by several layers of Pax2 positive cells (yellow) demonstrating cells organised as cap mesenchyme around developing structures at day six of culture in a spheroid made from re-aggregated E13.5 mouse kidney cells.
(AVI)

S2 Video PNA live staining of spheroid. Maximum intensity projection time series of a 4-day old spheroid labelled with PNA. The dye was added to the system upon the start of imaging. The imaging interval was 30 minutes. Initially, the spheroid is indiscriminately labelled but from the 4.0 hr imaging frame onwards, structures become cognisable. An arrow indicates a single labelled cell throughout the time series that integrates into a tubule at 8.5 hr.
(AVI)

S3 Video Time series of the maximum intensity projection of a live time series. Cells in green are expressing WT1-GFP, the red signal are basement membranes stained with PNA. The bright green structures are green beads that were dispersed throughout the agarose gel, providing fiduciary markers for multiview reconstruction of the time series.
(AVI)

S4 Video Detail of a live time series. Detail of the time series shown in S3 Video. Maximum intensity projection of a live time series. Cells in green are expressing WT1-GFP, the red signal are basement membranes stained with PNA. Several cells can be seen migrating within and around tubules labelled with PNA.
(AVI)

S5 Video Time series of the maximum intensity projection of a live time series. Cells in green are expressing WT1-GFP, the red signal are basement membranes stained with PNA. The bright green structures are green beads that were dispersed throughout the agarose gel, providing fiduciary markers for multiview reconstruction of the time series.
(AVI)

S6 Video Spot distribution of “Experiment 1" as determined by the automated tracking tool TrackMate. (AVI)

## References

[1] Grobstein C. Trans-filter induction of tubules in mouse metanephrogenic mesenchyme. Experimental Cell Research. 1956;10(2):424–40. 10.1016/0014-4827(56)90016-7. 13317909

[2] Rak-Raszewska A, Vainio S. Nephrogenesis in organoids to develop novel drugs and progenitor cell based therapies. European Journal of Pharmacology. 2016;790:3–11. 10.1016/j.ejphar.2016.07.011. 27395798

[3] Lanza RP, Chung HY, Yoo JJ, Wettstein PJ, Blackwell C, Borson N, et al Generation of histocompatible tissues using nuclear transplantation. Nat Biotech. 2002;20(7):689–96. 10.1038/nbt703 12089553

[4] Sebinger DDR, Unbekandt M, Ganeva VV, Ofenbauer A, Werner C, Davies JA. A Novel, Low-Volume Method for Organ Culture of Embryonic Kidneys That Allows Development of Cortico-Medullary Anatomical Organization. PLoS ONE. 2010;5(5):e10550 10.1371/journal.pone.0010550 20479933 PMC2866658

[5] Unbekandt M, Davies JA. Dissociation of embryonic kidneys followed by reaggregation allows the formation of renal tissues. Kidney Int. 2009;77(5):407–16. http://www.nature.com/ki/journal/v77/n5/suppinfo/ki2009482s1.html. 10.1038/ki.2009.482 20016472

[6] Rak-Raszewska A, Wilm B, Edgar D, Kenny S, Woolf AS, Murray P. Development of embryonic stem cells in recombinant kidneys. Organogenesis. 2012;8(4):125–36. 10.4161/org.22597 23086378 PMC3562253

[7] Ranghini E, Mora CF, Edgar D, Kenny SE, Murray P, Wilm B. Stem Cells Derived from Neonatal Mouse Kidney Generate Functional Proximal Tubule-Like Cells and Integrate into Developing Nephrons In Vitro. PLoS ONE. 2013;8(5):e62953 10.1371/journal.pone.0062953 23667549 PMC3646983

[8] Kuzma-Kuzniarska M, Rak-Raszewska A, Kenny S, Edgar D, Wilm B, Fuente Mora C, et al Integration potential of mouse and human bone marrow-derived mesenchymal stem cells. Differentiation. 2012;83(3):128–37. 10.1016/j.diff.2011.11.004. 22364880

[9] Siegel N, Rosner M, Unbekandt M, Fuchs C, Slabina N, Dolznig H, et al Contribution of human amniotic fluid stem cells to renal tissue formation depends on mTOR. Human Molecular Genetics. 2010;19(17):3320–31. 10.1093/hmg/ddq236 20542987

[10] Xinaris C, Benedetti V, Rizzo P, Abbate M, Corna D, Azzollini N, et al In Vivo Maturation of Functional Renal Organoids Formed from Embryonic Cell Suspensions. Journal of the American Society of Nephrology. 2012 10.1681/asn.2012050505 23085631 PMC3482737

[11] Dressler GR. The Cellular Basis of Kidney Development. Annual Review of Cell and Developmental Biology. 2006;22(1):509–29. 10.1146/annurev.cellbio.22.010305.104340 . 16822174

[12] Little MH, McMahon AP. Mammalian Kidney Development: Principles, Progress, and Projections. Cold Spring Harbor Perspectives in Biology. 2012;4(5). 10.1101/cshperspect.a008300 22550230 PMC3331696

[13] Taguchi A, Kaku Y, Ohmori T, Sharmin S, Ogawa M, Sasaki H, et al Redefining the In Vivo Origin of Metanephric Nephron Progenitors Enables Generation of Complex Kidney Structures from Pluripotent Stem Cells. Cell Stem Cell. 2014;14(1):53–67. 10.1016/j.stem.2013.11.010. 24332837

[14] Rak-Raszewska A, Hauser PV, Vainio S. Organ In Vitro Culture: What Have We Learned about Early Kidney Development? Stem Cells International. 2015;2015:16 10.1155/2015/959807 26078765 PMC4452498

[15] Lusis M, Li J, Ineson J, Christensen ME, Rice A, Little MH. Isolation of clonogenic, long-term self renewing embryonic renal stem cells. Stem Cell Research. 2010;5(1):23–39. 10.1016/j.scr.2010.03.003. 20434421

[16] Riccio P, Cebrian C, Zong H, Hippenmeyer S, Costantini F. Ret and Etv4 Promote Directed Movements of Progenitor Cells during Renal Branching Morphogenesis. PLOS Biology. 2016;14(2):e1002382 10.1371/journal.pbio.1002382 26894589 PMC4760680

[17] Lindström NO, Lawrence ML, Burn SF, Johansson JA, Bakker ERM, Ridgway RA, et al Integrated β-catenin, BMP, PTEN, and Notch signalling patterns the nephron. eLife. 2015;4:e04000 10.7554/eLife.04000 25647637 PMC4337611

[18] Saarela U, Akram SU, Desgrange A, Rak-Raszewska A, Shan J, Cereghini S, et al Novel fixed Z-dimension (FiZD) kidney primordia and an organoid culture system for time-lapse confocal imaging. Development. 2017 10.1242/dev.142950 28219945 PMC5358112

[19] Takasato M, Er PX, Becroft M, Vanslambrouck JM, Stanley EG, Elefanty AG, et al Directing human embryonic stem cell differentiation towards a renal lineage generates a self-organizing kidney. Nat Cell Biol. 2014;16(1):118–26. 10.1038/ncb2894 http://www.nature.com/ncb/journal/v16/n1/abs/ncb2894.html#supplementary-information. 24335651

[20] Huisken J, Swoger J, Del Bene F, Wittbrodt J, Stelzer EHK. Optical Sectioning Deep Inside Live Embryos by Selective Plane Illumination Microscopy. Science. 2004;305(5686):1007–9. 10.1126/science.1100035 15310904

[21] Kaufmann A, Mickoleit M, Weber M, Huisken J. Multilayer mounting enables long-term imaging of zebrafish development in a light sheet microscope. Development. 2012;139(17):3242–7. 10.1242/dev.082586 22872089

[22] Mertz J. Optical sectioning microscopy with planar or structured illumination. Nat Meth. 2011;8(10):811–9. 10.1038/nmeth.1709 21959136

[23] Pampaloni F, Chang B-J, Stelzer EK. Light sheet-based fluorescence microscopy (LSFM) for the quantitative imaging of cells and tissues. Cell and Tissue Research. 2015;360(1):129–41. 10.1007/s00441-015-2144-5 25743693

[24] Pampaloni F, Berge U, Marmaras A, Horvath P, Kroschewski R, Stelzer EHK. Tissue-culture light sheet fluorescence microscopy (TC-LSFM) allows long-term imaging of three-dimensional cell cultures under controlled conditions. Integrative Biology. 2014;6(10):988–98. 10.1039/c4ib00121d 25183478

[25] Pampaloni F, Richa R, Ansari N, Stelzer EK. Live Spheroid Formation Recorded with Light Sheet-Based Fluorescence Microscopy In: Verveer PJ, editor. Advanced Fluorescence Microscopy. Methods in Molecular Biology. 1251: Springer New York; 2015 p. 43–57. 10.1007/978-1-4939-2080-8_3 25391793

[26] Lorenzo C, Frongia C, Jorand R, Fehrenbach J, Weiss P, Maandhui A, et al Live cell division dynamics monitoring in 3D large spheroid tumor models using light sheet microscopy. Cell Division. 2011;6(1):22 10.1186/1747-1028-6-22 22152157 PMC3274476

[27] Swoger J, Pampaloni F, Stelzer EHK. Imaging Cellular Spheroids with a Single (Selective) Plane Illumination Microscope. Cold Spring Harbor Protocols. 2014;2014(1):pdb.prot080176. 10.1101/pdb.prot080176 24371324

[28] Swoger J, Pampaloni F, Stelzer EHK. Imaging MDCK Cysts with a Single (Selective) Plane Illumination Microscope. Cold Spring Harbor Protocols. 2014;2014(1):114–8. 10.1101/pdb.prot080184 24371325

[29] Yamashita N, Morita M, Legant WR, Chen B-C, Betzig E, Yokota H, et al Three-dimensional tracking of plus-tips by lattice light-sheet microscopy permits the quantification of microtubule growth trajectories within the mitotic apparatus. Journal of Biomedical Optics. 2015;20(10):101206-. 10.1117/1.JBO.20.10.101206 26527322

[30] Strnad P, Gunther S, Reichmann J, Krzic U, Balazs B, de Medeiros G, et al Inverted light-sheet microscope for imaging mouse pre-implantation development. Nat Meth. 2016;13(2):139–42. 10.1038/nmeth.3690 http://www.nature.com/nmeth/journal/v13/n2/abs/nmeth.3690.html#supplementary-information. 26657559

[31] Krzic U, Gunther S, Saunders TE, Streichan SJ, Hufnagel L. Multiview light-sheet microscope for rapid in toto imaging. Nat Meth. 2012;9(7):730–3. http://www.nature.com/nmeth/journal/v9/n7/abs/nmeth.2064.html#supplementary-information. 10.1038/nmeth.2064 22660739

[32] Keller PJ, Schmidt AD, Wittbrodt J, Stelzer EHK. Reconstruction of Zebrafish Early Embryonic Development by Scanned Light Sheet Microscopy. Science. 2008;322(5904):1065–9. 10.1126/science.1162493 18845710

[33] Henry KS, Wieland WH, Powell DE, Giesy JP. Laboratory analyses of the potential toxicity of sediment-associated polydimethylsiloxane to benthic macroinvertebrates. Environmental Toxicology and Chemistry. 2001;20(11):2611–6. 10.1002/etc.5620201129 11699789

[34] Hosen N, Shirakata T, Nishida S, Yanagihara M, Tsuboi A, Kawakami M, et al The Wilms’ tumor gene WT1-GFP knock-in mouse reveals the dynamic regulation of WT1 expression in normal and leukemic hematopoiesis. Leukemia. 2007;21(8):1783–91. 10.1038/sj.leu.2404752 17525726

[35] Chung K, Deisseroth K. CLARITY for mapping the nervous system. Nat Meth. 2013;10(6):508–13. 10.1038/nmeth.2481 23722210

[36] Chung K, Wallace J, Kim S-Y, Kalyanasundaram S, Andalman AS, Davidson TJ, et al Structural and molecular interrogation of intact biological systems. Nature. 2013;497(7449):332–7. 10.1038/nature12107 http://www.nature.com/nature/journal/v497/n7449/abs/nature12107.html#supplementary-information. 23575631 PMC4092167

[37] Poguzhelskaya E, Artamonov D, Bolshakova A, Vlasova O, Bezprozvanny I. Simplified method to perform CLARITY imaging. Molecular Neurodegeneration. 2014;9(1):19 10.1186/1750-1326-9-19 24885504 PMC4049387

[38] Tomer R, Ye L, Hsueh B, Deisseroth K. Advanced CLARITY for rapid and high-resolution imaging of intact tissues. Nat Protocols. 2014;9(7):1682–97. 10.1038/nprot.2014.123 http://www.nature.com/nprot/journal/v9/n7/abs/nprot.2014.123.html#supplementary-information. 24945384 PMC4096681

[39] Schindelin J, Arganda-Carreras I, Frise E, Kaynig V, Longair M, Pietzsch T, et al Fiji: an open-source platform for biological-image analysis. Nat Meth. 2012;9(7):676–82. http://www.nature.com/nmeth/journal/v9/n7/abs/nmeth.2019.html#supplementary-information. 10.1038/nmeth.2019 PMC3855844 22743772

[40] Jaqaman K, Loerke D, Mettlen M, Kuwata H, Grinstein S, Schmid SL, et al Robust single-particle tracking in live-cell time-lapse sequences. Nature Methods. 2008;5:695 10.1038/nmeth.1237 https://www.nature.com/articles/nmeth.1237#supplementary-information. 18641657 PMC2747604

[41] Schmid B, Schindelin J, Cardona A, Longair M, Heisenberg M. A high-level 3D visualization API for Java and ImageJ. BMC Bioinformatics. 2010;11(1):274 10.1186/1471-2105-11-274 20492697 PMC2896381

[42] Tinevez J-Y, Perry N, Schindelin J, Hoopes GM, Reynolds GD, Laplantine E, et al TrackMate: An open and extensible platform for single-particle tracking. Methods. 2017;115:80–90. 10.1016/j.ymeth.2016.09.016. 27713081

[43] Preibisch S, Saalfeld S, Schindelin J, Tomancak P. Software for bead-based registration of selective plane illumination microscopy data. Nat Meth. 2010;7(6):418–9. http://www.nature.com/nmeth/journal/v7/n6/suppinfo/nmeth0610-418_S1.html. 10.1038/nmeth0610-418 20508634

[44] Icha J, Schmied C, Sidhaye J, Tomancak P, Preibisch S, Norden C. Using Light Sheet Fluorescence Microscopy to Image Zebrafish Eye Development. 2016;(110):e53966 10.3791/53966 27167079 PMC4941907

[45] Davies J, Chang CH. Engineering kidneys from simple cell suspensions: an exercise in self-organization. Pediatric Nephrology. 2014;29(4):519–24. 10.1007/s00467-013-2579-4 23989397 PMC3928531

[46] Davies J, Unbekandt M, Ineson J, Lusis M, Little M. Dissociation of Embryonic Kidney Followed by Re-aggregation as a Method for Chimeric Analysis In: Michos O, editor. Kidney Development. Methods in Molecular Biology^™^. 886: Humana Press; 2012 p. 135–46. 10.1007/978-1-61779-851-1_12 22639257

[47] Lefevre JG, Chiu HS, Combes AN, Vanslambrouck JM, Ju A, Hamilton NA, et al Self-organisation after embryonic kidney dissociation is driven via selective adhesion of ureteric epithelial cells. Development. 2017;144(6):1087–96. 10.1242/dev.140228 28174247

[48] Parreira KS, Debaix H, Cnops Y, Geffers L, Devuyst O. Expression patterns of the aquaporin gene family during renal development: influence of genetic variability. Pflügers Archiv—European Journal of Physiology. 2009;458(4):745–59. 10.1007/s00424-009-0667-x 19367412 PMC2756349

[49] Basson MA, Akbulut S, Watson-Johnson J, Simon R, Carroll TJ, Shakya R, et al Sprouty1 Is a Critical Regulator of GDNF/RET-Mediated Kidney Induction. Developmental Cell. 2005;8(2):229–39. 10.1016/j.devcel.2004.12.004. 15691764

[50] Junttila S, Saarela U, Halt K, Manninen A, Pärssinen H, Lecca MR, et al Functional Genetic Targeting of Embryonic Kidney Progenitor Cells Ex Vivo. Journal of the American Society of Nephrology. 2015;26(5):1126–37. 10.1681/ASN.2013060584 25201883 PMC4413750

[51] Torres M, Gomez-Pardo E, Dressler GR, Gruss P. Pax-2 controls multiple steps of urogenital development. Development. 1995;121(12):4057–65. 8575306 10.1242/dev.121.12.4057

[52] Wilm B, Muñoz-Chapuli R. The Role of WT1 in Embryonic Development and Normal Organ Homeostasis In: Hastie N, editor. The Wilms’ Tumor (WT1) Gene: Methods and Protocols. New York, NY: Springer New York; 2016 p. 23–39. 10.1007/978-1-4939-4023-3_3 27417957

[53] Kreidberg JA. WT1 and kidney progenitor cells. Organogenesis. 2010;6(2):61–70. 10.4161/org.6.2.11928 20885852 PMC2901809

[54] Harding SD, Armit C, Armstrong J, Brennan J, Cheng Y, Haggarty B, et al The GUDMAP database—an online resource for genitourinary research. Development. 2011;138(13):2845–53. 10.1242/dev.063594 21652655 PMC3188593

[55] McMahon AP, Aronow BJ, Davidson DR, Davies JA, Gaido KW, Grimmond S, et al GUDMAP: The Genitourinary Developmental Molecular Anatomy Project. Journal of the American Society of Nephrology. 2008;19(4):667–71. 10.1681/ASN.2007101078 18287559

[56] Quaggin SE, Kreidberg JA. Development of the renal glomerulus: good neighbors and good fences. Development. 2008;135(4):609–20. 10.1242/dev.001081 18184729

[57] Lawrence ML, Chang CH, Davies JA. Transport of organic anions and cations in murine embryonic kidney development and in serially-reaggregated engineered kidneys. Scientific Reports. 2015;5:9092 10.1038/srep09092 http://www.nature.com/articles/srep09092#supplementary-information. 25766625 PMC4357899

