# Supplementary figures and images for: Ex vivo live cell tracking in kidney organoids using light sheet fluorescence microscopy
Source: PLoS One. 2018 Jul 26;13(7):e0199918. doi: 10.1371/journal.pone.0199918 (PMC6062017; doi:10.1371/journal.pone.0199918)

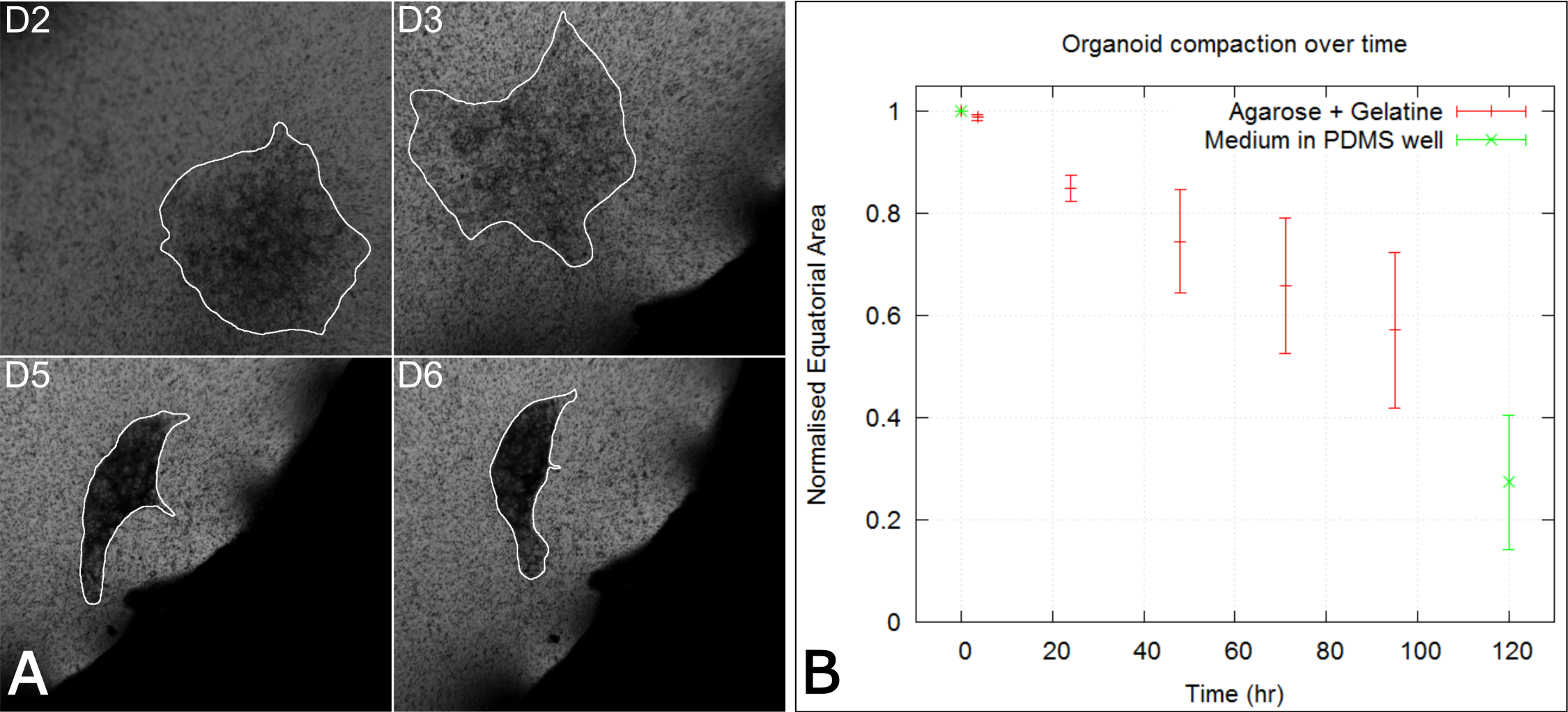

Supplement: S1 Fig — A) Compaction and increasing optical density of a pellet cultured at air-liquid interface. The pellet was incubated on a micro-porous membrane and the label in the top left corner indicates the day of culture. At day two and three, the pellet has a comparatively large footprint (white outline) on the membrane whereas at day five, the cells have self assembled into a tissue with a much smaller footprint that appears much darker with some visible tubule-like structures. B) Organoid compaction over time measured as the equatorial area of organoids over five days. The agarose + gelatine refers to organoids that were embedded into this gel mixture 24 hours after re-aggregation. Error bars are the standard deviation of the normalised data. (TIF) [file pone.0199918.s001.tif]

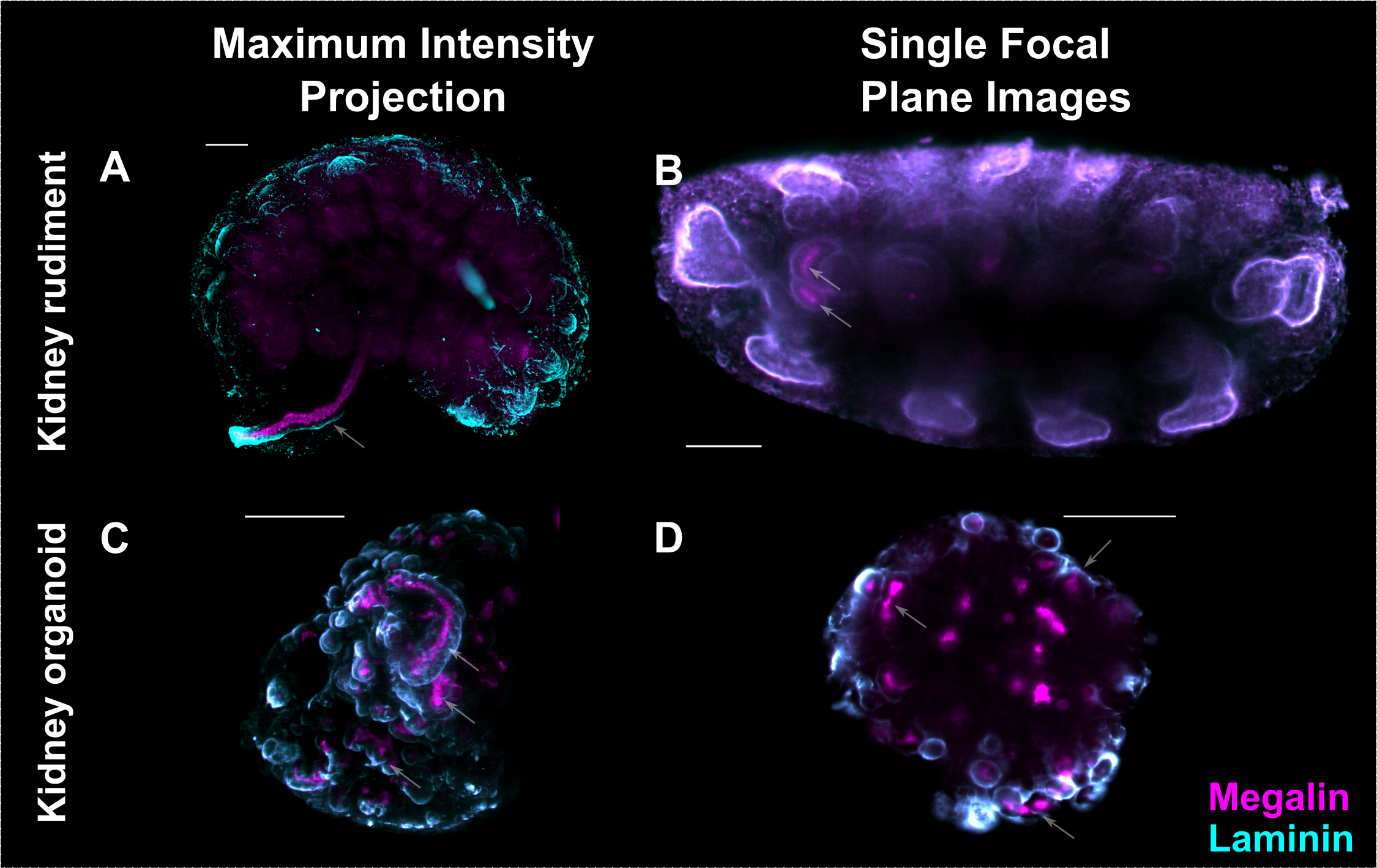

Supplement: S2 Fig — (A) shows the maximum intensity projection and (B) a single focal plane of cleared whole mount fixed and stained mouse embryonic E13.5 kidneys. There are few megalin+ structures in the whole rudiments (arrows), representative of the early developmental stage of the kidneys where only few tubules have developed. (B) shows large laminin+ structures which are megalin-, indicative of the ureteric bud. (C) shows the maximum intensity projection and (D) a single focal plane of a cleared organoid that was fixed after six days of culture. There are many megalin+ structures, surrounded by laminin+ membranes, indicative of nephric tubules (arrows). For both, kidney rudiments and specifically organoids, the megalin penetration was superior to the laminin penetration and there are megalin+ structures that appear to be laminin-, which we consider untrue. Increasing the incubation times resulted in increased unspecific staining but not in improved penetration depth. Had the laminin stain penetrated deeper, we would have expected staining around those megalin+ lumens as well. All samples were cleared. Scale bars 100 μm. (TIF) [file pone.0199918.s002.tif]

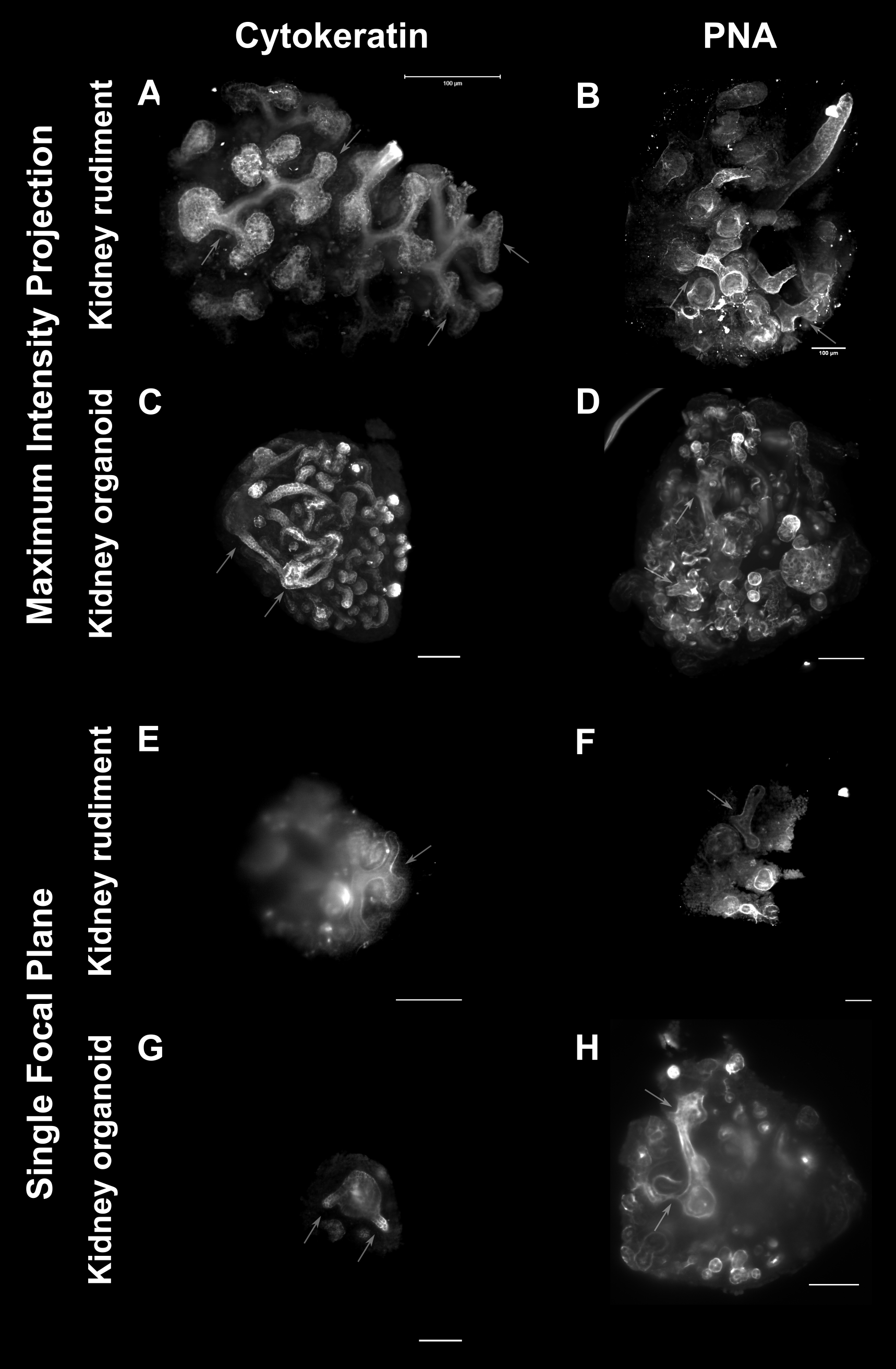

Supplement: S3 Fig — A-D show maximum intensity projections and E-H show single focal plane images of whole mount fixed and stained mouse embryonic E13.5 kidney rudiments and organoids that were cultured for six days before fixing. Both, the cytokeratin and PNA staining reveal branched structures (arrows) in the intact rudiments and the spheroids. In the intact kidney rudiment in (A), the cytokeratin+ cells are arranged in the typical tree shape of the ureteric bud, i.e. an intricate branch system connected to one base structure. The tree shape of another embryonic kidney is also highlighted by the basement membrane stain PNA in (B). In the spheroid in (C), the cytokeratin+ cells are not organised in an interconnected structure. There are many independent cytokeratin+ structures that have developed from different ureteric bud foci, causing a less organised ureteric tree structure, which is also reflected in the PNA staining, which simultaneously highlights developing nephrons. All samples but the spheroid shown in C/G were cleared. Scale bars 100 μm. (TIF) [file pone.0199918.s003.tif]

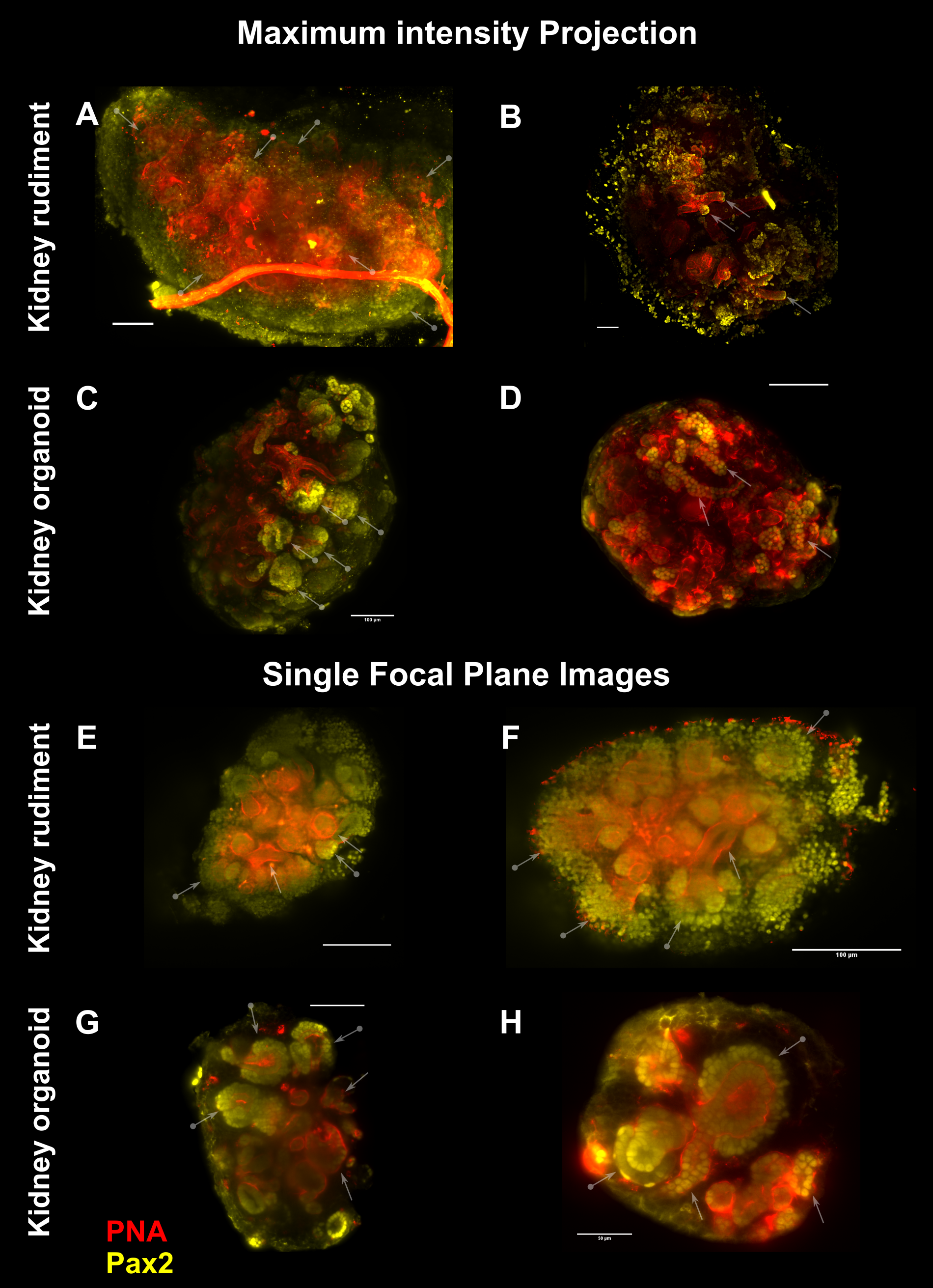

Supplement: S4 Fig — A-D show maximum intensity projections and E-H show single focal plane images of whole mount fixed and stained mouse embryonic E13.5 kidneys and organoids that were cultured for six days before fixing and staining. The Pax2+ cells are grouped around structures highlighted with PNA, i.e. cap mesenchyme (arrows with dot end). E-H show that the cap mesenchyme consists of 4–5 layers of cells, both in intact kidney rudiments and 6-day old organoids. Pax2+ cells are also present in structures surrounded by basement membrane, indicating ureteric bud (arrows). All samples were cleared. Scale bars 100 μm. (TIF) [file pone.0199918.s004.tif]

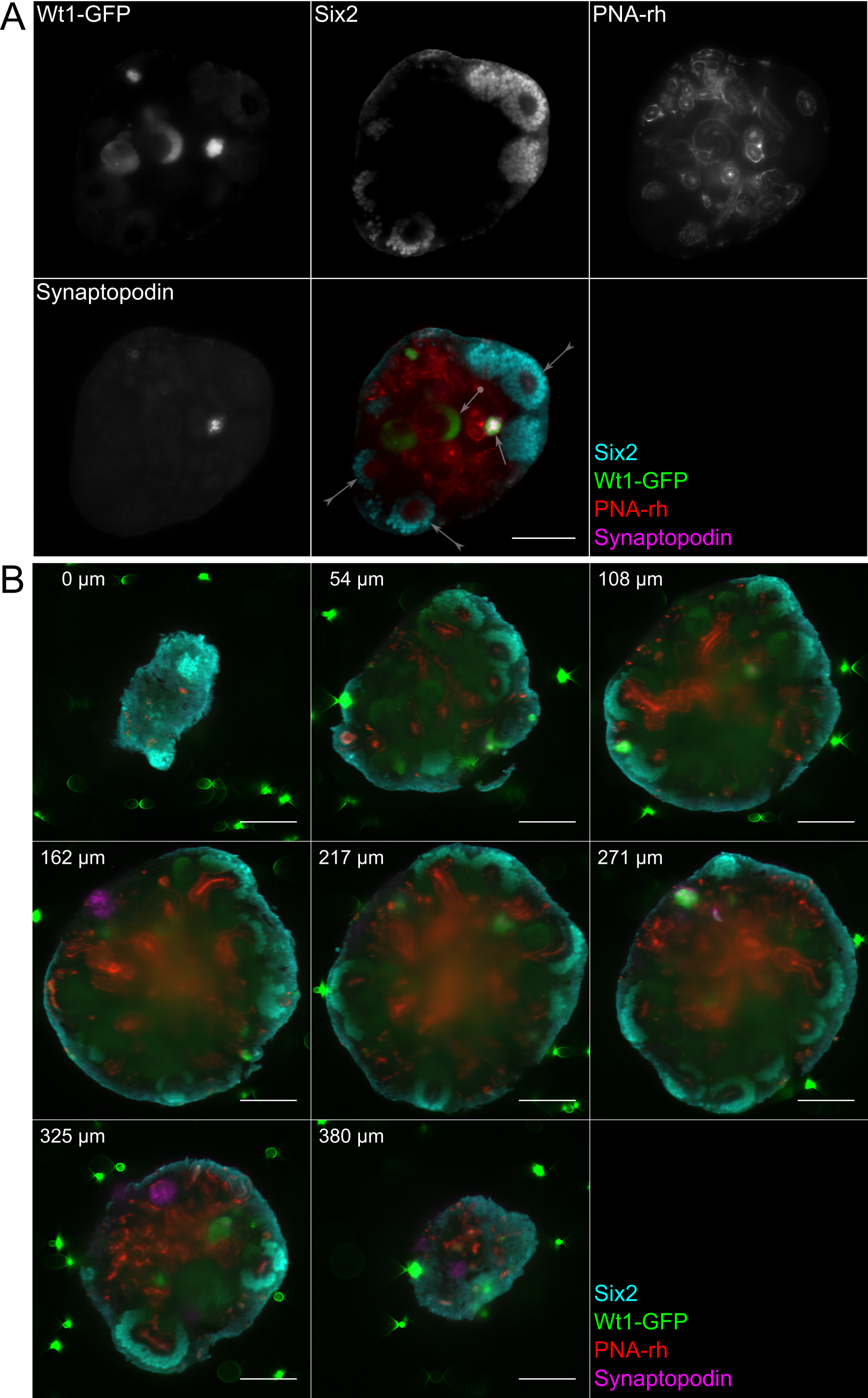

Supplement: S5 Fig — The images in (A) represent a single focal plane of a 6-day old renal organoid expressing Wt1-GFP and stained with PNA to label basement membranes, Six2 for cap mesenchyme and Synaptopodin for podocytes. The Six2+ cells are aligned around ureteric bud highlighted with PNA (arrows with arrow head ends) and also slightly express Wt1-GFP. In the centre of the spheroid, there is an s-shaped body (arrow with dot end), which contains cells expressing Wt1-GFP. A glomerulus (arrow) is located right of the s-shaped body and strongly expresses Wt1 and is also Synaptopodin+. This staining pattern indicates that there are mature renal structures and nascent tubules within the spheroid at the same time. (B) shows the same organoid but at different depth indicated in the left top corner of each frame. The PNA and Wt1 signal are strong throughout the whole depth but the Six2 signal is limited to the first 75 μm and unfortunately absent deep in the tissue. This organoid was not cleared. The organoid was recorded from five different angles and the data was successfully fused in Fiji. Scale bars: 100 μm. (TIF) [file pone.0199918.s005.tif]

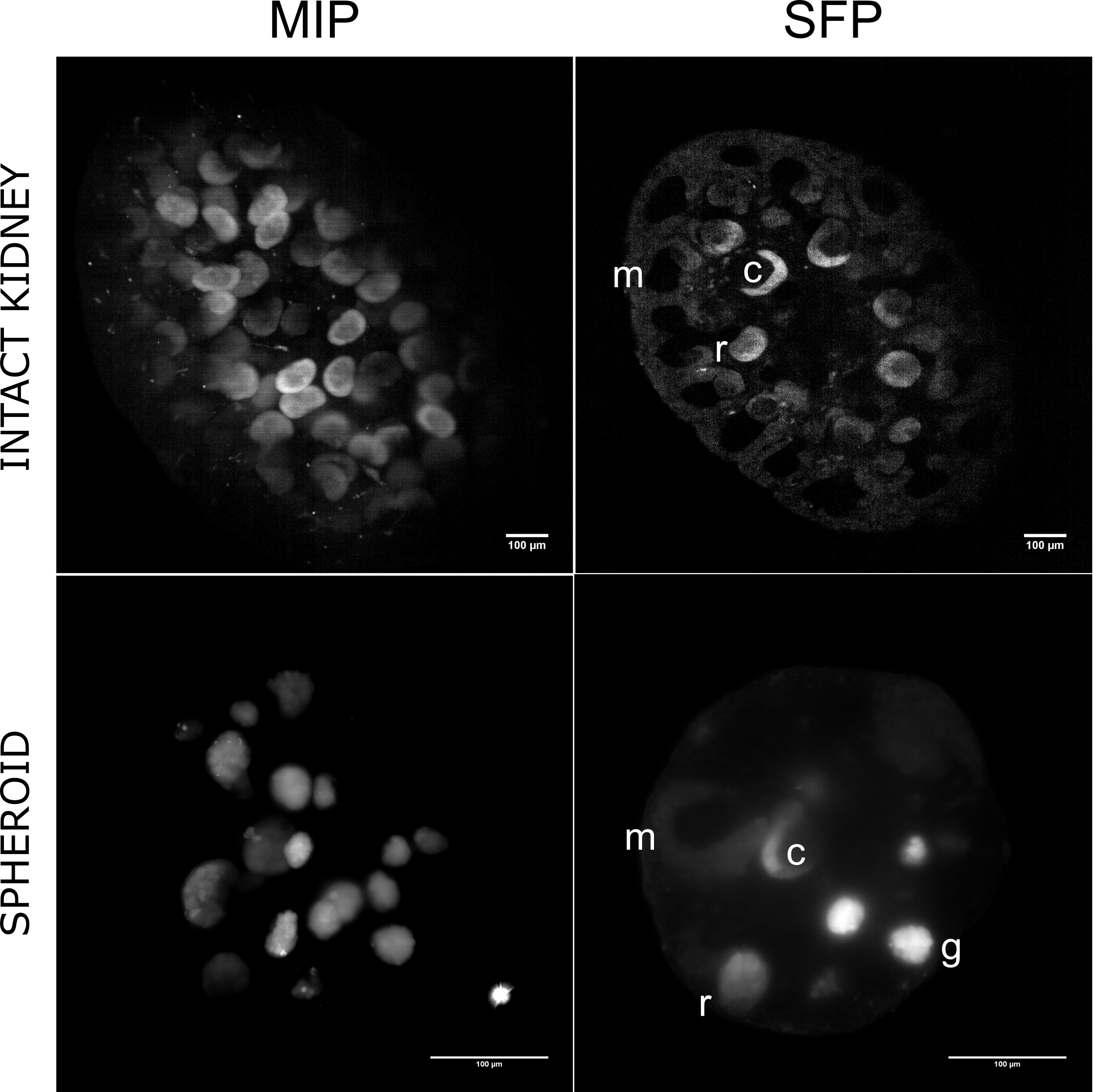

Supplement: S6 Fig — The same developmental structures—except glomerulus-like structures—can be identified in the in vivo and in vitro tissues: metanephric mesenchyme (m), cap mesenchyme (c), renal vesicle (r), glomerular structure (g). The Wt1-GFP signal was weak in the metanephric mesenchyme, increased in the cap-mesenchyme and further increased as the tubular and in particular the glomerular stages are reached. MIP: Maximum Intensity Projection, SFP: Single Focal Plane, Scale bar: 100 μm. (TIF) [file pone.0199918.s006.tif]

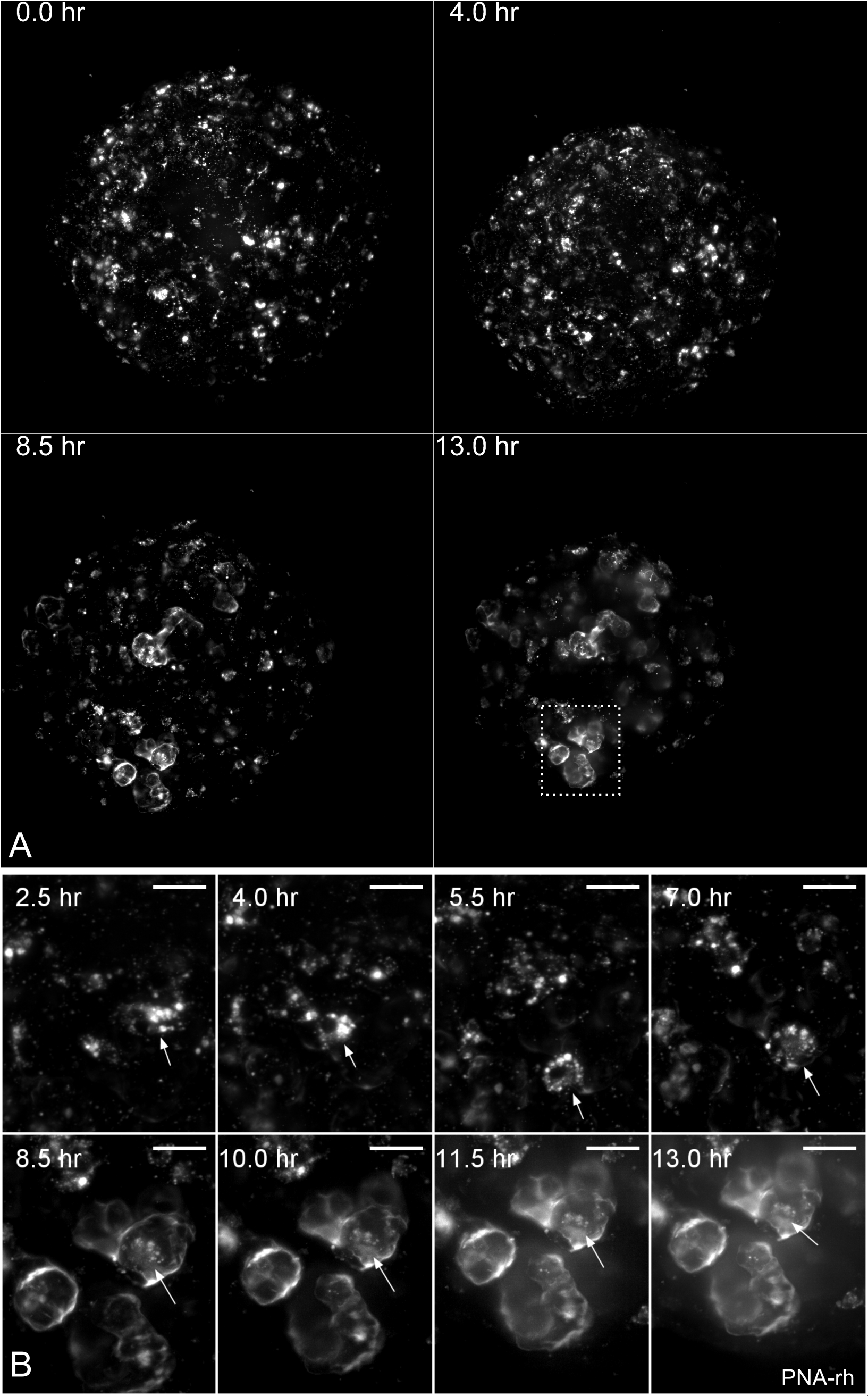

Supplement: S7 Fig — (A) maximum intensity projections of an organoid cultured for four days before embedding into a hydrogel cylinder, continued culture in the microscope and imaging (T = 0.0 hr). Over time, the PNA dye penetrated the organoid and tubular structures became visible. The dashed rectangle in the 13 hr panel highlights the area showing as a detailed view of the same time series in B. The movement of a single cell (arrow) migrating towards and integrating into a tubule at T = 8.5 hr can be followed throughout the course of the time series. Scale bar: 25μm. (TIF) [file pone.0199918.s007.tif]

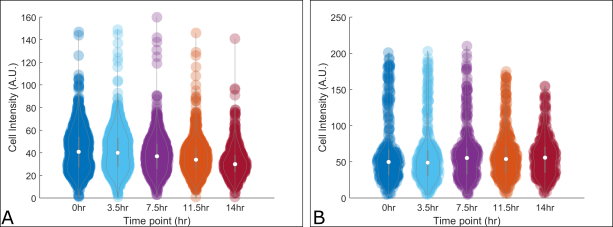

Supplement: S8 Fig — The distributions remain similar over time with a slight decrease of the mean in Times Series 1 (A) and a slight increase of the mean in Time Series 2 (B). (PNG) [file pone.0199918.s008.png]
